# A proteomic and functional view of intrabacterial lipid inclusion biogenesis in mycobacteria

**DOI:** 10.1128/mbio.01475-24

**Published:** 2025-02-25

**Authors:** Tonia Dargham, John Jairo Aguilera-Correa, Romain Avellan, Ivy Mallick, Léa Celik, Pierre Santucci, Gael Brasseur, Isabelle Poncin, Vanessa Point, Stéphane Audebert, Luc Camoin, Wassim Daher, Jean-François Cavalier, Laurent Kremer, Stéphane Canaan

**Affiliations:** 1 Aix-Marseille Univ., CNRS, LISM-UMR 7255, IMM FR3479, IM2B, Marseille, France; 2 IHU Méditerranée Infection, Aix-Marseille Univ., Marseille, France; 3 CNRS UMR 9004, IRIM, Université de Montpellier, Montpellier, France; 4 Aix-Marseille Univ., CNRS, LCB-UMR 7283, IMM FR3479, IM2B, Marseille, France; 5 Aix-Marseille Univ., INSERM, CNRS, Institut Paoli-Calmettes, CRCM, Marseille, France; 6 INSERM, IRIM 131821 , Montpellier, France; National University of Singapore, Singapore, Singapore

**Keywords:** Intracellular lipid inclusion, lipid metabolism, persistence, *Mycobacterium abscessus*, ILI-associated proteins, proteomic, proximity labeling, APEX2

## Abstract

During infection and granuloma formation, pathogenic mycobacteria store triacylglycerol as intrabacterial lipid inclusions (ILIs). This accumulation of nutrients provides a carbon source for bacterial persistence and slows down intracellular metabolism. *Mycobacterium abscessus* (*Mab*), a rapidly growing non-tuberculous actinobacterium, produces ILI throughout its infection cycle. Here, *Mab* was used as a model organism to identify proteins associated with ILI accumulation on a global scale. By using the APEX2 proximity labeling method in an *in vitro* model for ILI accumulation, we identified 228 proteins possibly implicated in ILI biosynthesis. Fluorescence microscopy of strains overexpressing eight ILI-associated proteins (IAP) candidates fused to superfolder green fluorescent protein showed co-localization with ILI. Genetic inactivation of these potential IAP-encoding genes and subsequent lipid analysis emphasized the importance of MAB_3486 and MAB_4532c as key enzymes influencing triacylglycerol storage. This study underscores the dynamic process of ILI biogenesis and advances our understanding of lipid metabolism in pathogenic mycobacteria. Identifying major IAP in lipid accumulation offers new therapeutic perspectives to control the growth and persistence of pathogenic mycobacteria.

**IMPORTANCE:**

This study sheds light into the complex process of intracellular lipid accumulation and storage in the survival and persistence of pathogenic mycobacteria, which is of clinical relevance. By identifying the proteins involved in the formation of intrabacterial lipid inclusions and revealing their impact on lipid metabolism, our data may lead to the development of new therapeutic strategies to target and control pathogenic mycobacteria, potentially improving outcomes for patients with mycobacterial infections.

## INTRODUCTION

Actinobacteria accumulate neutral lipids as intrabacterial lipid inclusions (ILIs) under certain environmental conditions. Unlike many microorganisms that store glycogen, polyphosphates, and polyhydroxyalkanoates, mycobacteria store triacylglycerol (TAG) in their cytoplasm during particular stress conditions (1–4). During granuloma formation in *Mycobacterium tuberculosis* (*Mtb*) infected hosts, foamy macrophages accumulate large lipid bodies, which the bacilli use to synthesize ILI, enabling the pathogen to persist in a non-replicating dormant state for extended periods of time (5). ILI plays a crucial role in the virulence and induction of dormancy of *Mtb* (3, 6) and is associated with antibiotic tolerance (6, 7). All mycobacterial species can accumulate ILI in their cytoplasm, including *Mycobacterium abscessus* (*Mab*) (8), *Mycobacterium ulcerans* (9), *Mycobacterium avium* (10), *Mycobacterium bovis* BCG (*Mbv* BCG) (11, 12), and *Mycobacterium leprae* (13). ILI serves as an energy reservoir and plays a role in mycobacterial virulence, as shown by higher mortality rates in zebrafish infected with lipid-rich *Mab* (3, 8). Similarly, during tissue repair following *M. ulcerans* infection, bacilli adapt by modifying their metabolic activity and nutrient sources for survival in healing tissues (9). Cells infected with *M. leprae* also highlight the importance of ILI in pathogenesis, using host lipids for survival and inducing an inflammatory response conducive to bacterial proliferation (13). ILI has also been reported in saprophytic *Mycobacterium smegmatis* (*Msmeg*) since the 1970s (14, 15), and more recently, in *Mycobacterium brumae* (16), suggesting that these organelles confer significant advantages to the mycobacterial life cycle.

The role of proteins in ILI formation, function, and degradation remains poorly understood. Proteomic experiments on lipid droplets from *Rhodococcus opacus* (17) and *Rhodococcus jostii* (18) identified approximately 200 distinct proteins. In mycobacteria, only two studies had been conducted previously. The first one, using *Mbv* BCG (11), identified five proteins exclusively associated with ILI, while a second study using *Msmeg* (14) acknowledged around 400 potential ILI-associated proteins (IAP). A major difference between these studies lies in the ILI accumulation and purification methods employed, leading to variations in the ILI size and in the number of IAPs identified. Among the latter, a bifunctional enzyme with a synthase domain in its C-terminus and a lipase domain in its N-terminus was isolated among the 50 most abundant proteins in *Msmeg* (MSMEG_3637) or *Mbv* BCG (BCG1721), resulting in increased TAG production and degradation when overexpressed (11). Other IAP includes triglyceride synthase 1 (Tgs1), involved in TAG biosynthesis in *Mbv* BCG (11), *Mtb* (19), and *Mab* (8), while deletion of its gene drastically decreased TAG production. These two studies suggest a link between ILI accumulation/degradation and the action of both Tgs1 and the bifunctional enzyme long-chain fatty acid-CoA ligase (ACSL).

To gain insights into the ILI-protein interactions and the biological significance of this organelle in bacteria, an *in silico* analysis was conducted to identify a conserved ILI proteome (ILIome) core in Actinobacteria, using three independent ILI-associated proteomes (20). These bioinformatics findings revealed a conserved amphipathic helix in both Tgs1 and ACSL, potentially interacting with ILI (20). From these observations, it was inferred that Tgs1 and ACSL are highly conserved across all mycobacterial species and could serve as probes to identify neighboring partners involved in ILI synthesis and/or degradation.

This study aimed to define the ILIome involved in ILI formation and to elucidate the dynamics of this process. Unlike prior studies in *Mbv* BCG (11) and *Msmeg* (14), our approach employed a non-invasive method applied to living mycobacteria, based on the APEX2-proximity labeling technique. This method, combined with enrichment procedures, was designed to define the ILIome while minimizing false-positive hits caused by contamination with highly hydrophobic proteins. Indeed, in the presence of hydrogen peroxide, the APEX2 ascorbate peroxidase catalyzes the oxidation of a biotin-phenol substrate into a short-lived biotin-phenoxyl radical, which reacts with electron-rich amino acids of nearby proteins within a radius of less than 20 nm. The biotinylated proteins can then be enriched by streptavidin beads and identified by mass spectrometry (MS). APEX2 has already been successfully applied in mycobacteria to identify bacterial proteins specifically located in the cytoplasm or as specific substrates of the type VII secretion system (21–23). Compared to conventional methods like the two-hybrid system or co-immunoprecipitation coupled with MS, proximity labeling offers a robust approach for proteomic mapping (24–26), aiding in deciphering the kinetics of the ILIome in live cells by reducing potential artifacts.

Here, APEX2 (21, 25, 27) was fused to the ILI-associated protein Tgs1, and the resulting strain was used to monitor the ILIome kinetics, leading to the identification of 228 potential IAP, of which 100 are potentially involved in lipid biosynthetic pathways. Although additional studies are warranted to confirm the function and contribution of each IAP during ILI biogenesis, we validated our approach by focusing on 10 selected targets. This study reports the first comprehensive ILI proxisome thus providing new insight into the dynamics of ILI formation and paving the way for the discovery of potential therapeutic targets with translational value.

## RESULTS AND DISCUSSION

### Whole proteome analysis alone is insufficient to fully depict the ILIome content

**Fig 1:**
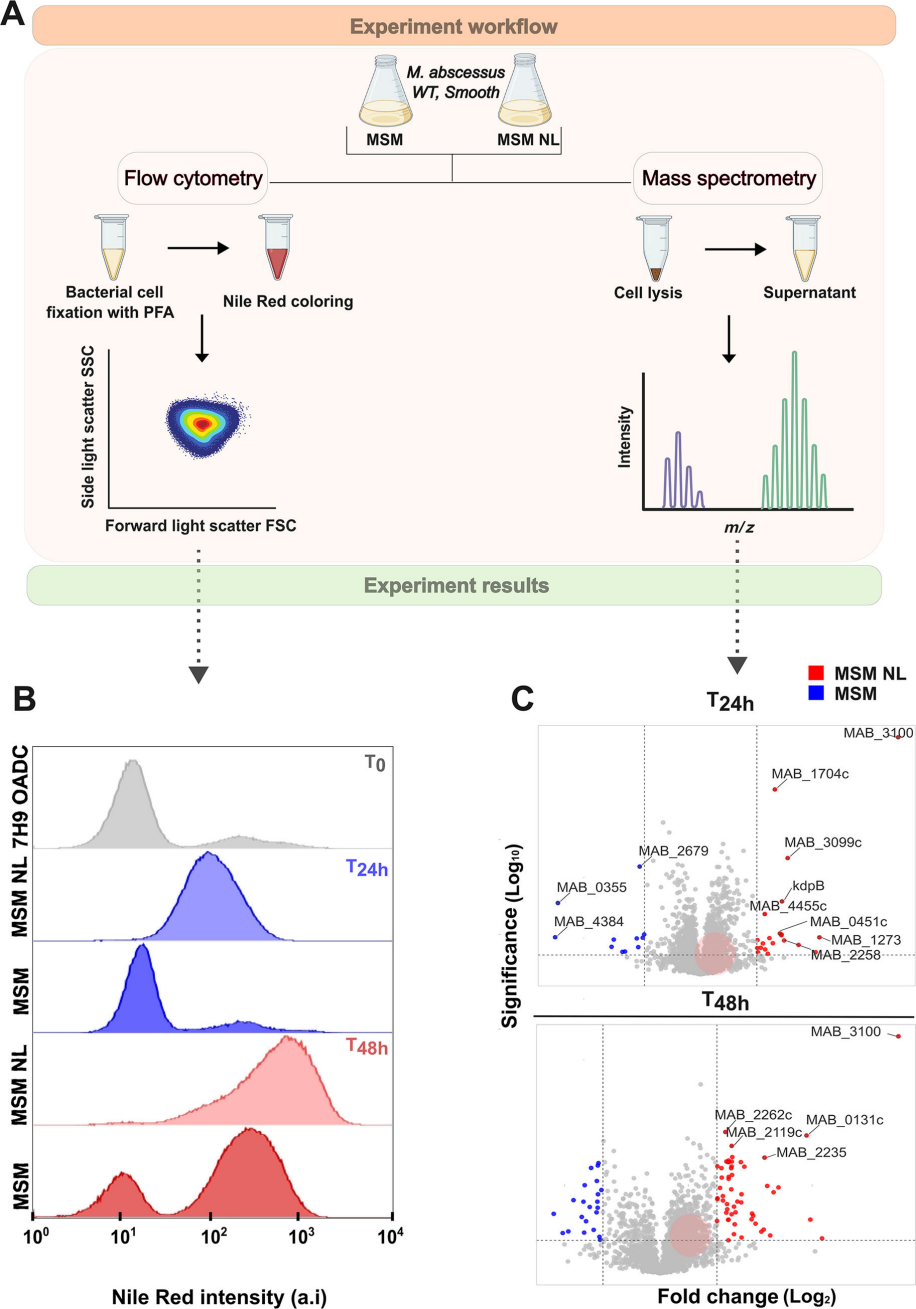
Whole proteome analysis. (**A**) Illustration depicting the whole proteome analysis workflow, including MS and flow cytometry. (**B**) Nile red fluorescence in *Mab* measured by flow cytometry: bacterial cells were harvested at 24 and 48 h from MSM and MSM NL, fixed and stained by Nile red, washed, and diluted in PBS. A total of 300,000 events were measured for each biological sample, and Nile Red fluorescence was collected at 615/25 nm. (**C**) Volcano map illustrating differentially expressed proteins. Blue dots represent up-regulated proteins in MSM, red dots indicate up-regulated proteins in MSM NL, and gray dots represent proteins with no significant difference, at 24 and 48 h of incubation. Red circles highlight lipid metabolism proteins. Generated using the VolcaNoseR web app (28).

To identify proteins involved in ILI biosynthesis, we used a previously established *in vitro* model employing a nitrogen-limiting minimal salt medium (MSM NL; Fig. 1A) (3). Initially, we assessed population homogeneity over time within this medium. Flow cytometry analysis after Nile Red staining enabled us to quantify lipid-associated fluorescent signals and distinguish between ILI-positive and ILI-negative bacteria (Fig. 1A). To ensure homogeneity of lipid-rich bacilli, flow cytometry analyzed bacterial populations at 24 and 48 h under two media conditions: MSM and MSM NL. MSM NL yielded a homogeneous population with a unimodal Gaussian distribution at both time points, indicating lipid-rich ILI-positive bacteria. Conversely, control MSM consistently displayed a bimodal Gaussian distribution, reflecting a diverse population of lipid-poor and lipid-rich bacteria (Fig. 1B). After confirming population homogeneity in MSM NL, we adapted the same culture workflow to isolate bacteria at 24 and 48 h for subsequent proteomic analyses, depicting the proteomic landscape during various ILI formation stages (Fig. 1C).

Given the significant lipid accumulation at 24 and 48 h in MSM NL, we initially conducted a full proteome analysis, anticipating an abundance of proteins associated with lipid metabolism. At 24 h, label-free proteomic analysis revealed 21 differentially expressed proteins in MSM NL compared to MSM (*P*-value ≤ 0.05 and a fold change [Log_2_] ≥1; Fig. 1C; Table S1). Among these proteins, five proteins (MAB_1066, MAB_1774, MAB_1262, MAB_4558c, and MAB_1704c) lack orthologs in *Mtb*, while only three proteins (MAB_4455c, MAB_2258, and MAB_2035) belong to the lipid metabolism functional category.

The remaining proteins are associated with cell wall processes (MAB_2448, MAB_1432, MAB_4615, MAB_1262, and MAB_0356c), intermediary metabolism (MAB_1273, MAB_0451 c, and MAB_0459c), virulence and detoxification processes (MAB_3904), information pathways (MAB_3100), or regulatory proteins (MAB_3099c).

At 48 h, a larger number of proteins was overproduced in both media compared to the 24 h time point (Fig. 1C; Table S2). Fifty-five proteins were more abundant in MSM NL, while 22 were over-produced in MSM. The most abundantly produced protein at 48 h was MAB_3100, an L-alanine dehydrogenase (Ald) involved in amino acid metabolism, also detected at 24 h. During *Mtb’s* non-replicating state, the alanine dehydrogenase Rv2780, sharing 77.8% sequence identity with MAB_3100, is upregulated, likely maintaining the optimal NADH/NAD ratio during anaerobiosis (29). Hence, the observed increase in MAB_3100 expression may suggest that lipid-rich bacteria may have entered a persistence-like physiological state, characterized by adaptations to environmental stresses, metabolic pathway alterations, and upregulation of specific genes, such as *ald* (29). Highly abundant proteins in MSM NL, including the ABC transporter MAB_2262c, the hypothetical protein MAB_0131c, the siderophore-interacting protein MAB_2235, and the putative polyketide synthase MAB_2119c involved in mycobactin synthesis (30), may be overproduced in response to iron limitation in the medium. Upregulation of these proteins may optimize iron acquisition under nitrogen-limiting conditions. Five proteins (MAB_3100, MAB_1704, MAB_3099, MAB_0459, and KdpB MAB_3256c), involved in secondary metabolism or classified as regulatory proteins, were present at both time points. Interestingly, the proteins identified by Low et al. (11) were present in our model at 48 h (coinciding with complete ILI formation) but not at 24 h, although not exhibiting significant differential expression levels. In contrast, among the 50 highly abundant proteins identified by Armstrong et al. in *Msmeg* (14), two proteins lack an ortholog in *Mab* (MSMEG_6291 and MSMEG_6049), while 12 proteins were undetected at 24 and 48 h (MSMEG_1030, MSMEG_1442, MSMEG_0098, MSMEG_4936, MSMEG_1445, MSMEG_2776, MSMEG_0023, MSMEG_1399, MSMEG_5430, MSMEG_1401, MSMEG_1368, and MSMEG_4938). These latter proteins are mainly involved in genetic information processing, including elongation factors or ribosomal subunits, or related to cofactor metabolism and ATP synthesis. The remaining proteins were detected in our model at both time points.

The identification of eight ribosomal proteins (rplP,rplO, rpsL, rpsK, rpsN, rspR, and rpmG1) in MSM suggests an increased demand for protein translation, possibly reflecting an adaptive metabolic response. The 11 remaining proteins are mainly conserved hypotheticals with unknown function.

Proteins involved in TAG biosynthesis pathways and lipid synthesis based on *Mtb* annotation were found in our data, with a fold change (Log_2_) between −0.5 and 0.5 and a -Log_10_ (*P-*value) less than 2 (Fig. 1C). We could only identify mycobactin and polyketide synthases. Furthermore, the near absence of TAG biosynthesis enzymes suggests that these pathways might not be the primary metabolic pathway occurring under these experimental conditions. It is plausible that other metabolic pathways are prioritized to persist in the nitrogen-limiting environment, emphasizing the complexity of bacterial metabolic responses and their adaptability to environmental cues.

These results support the view that ILI has broader functions, beyond energy storage. The identification of diverse protein families in our experimental design implies an underrepresentation of enzymes associated with lipid metabolism in our model. We suppose that non-physiological models can induce proteome bias, motivating the development of a more sensitive and specific technique, such as proximity labeling of the ILI core-structure using the APEX2 technology (22). Enriching proteins in the vicinity of the ILI is expected to offer a more accurate view of the ILIome, revealing specific key players associated with lipid metabolism during the formation of these organelles. Moreover, this approach could enhance our understanding of lipid metabolism dynamics and intricacies within mycobacterial cells *in vivo* and provide a more accurate depiction of the metabolic landscape during ILI synthesis.

### Validation of APEX2 proximity labeling for identifying ILI-associated proteins

**Fig 2:**
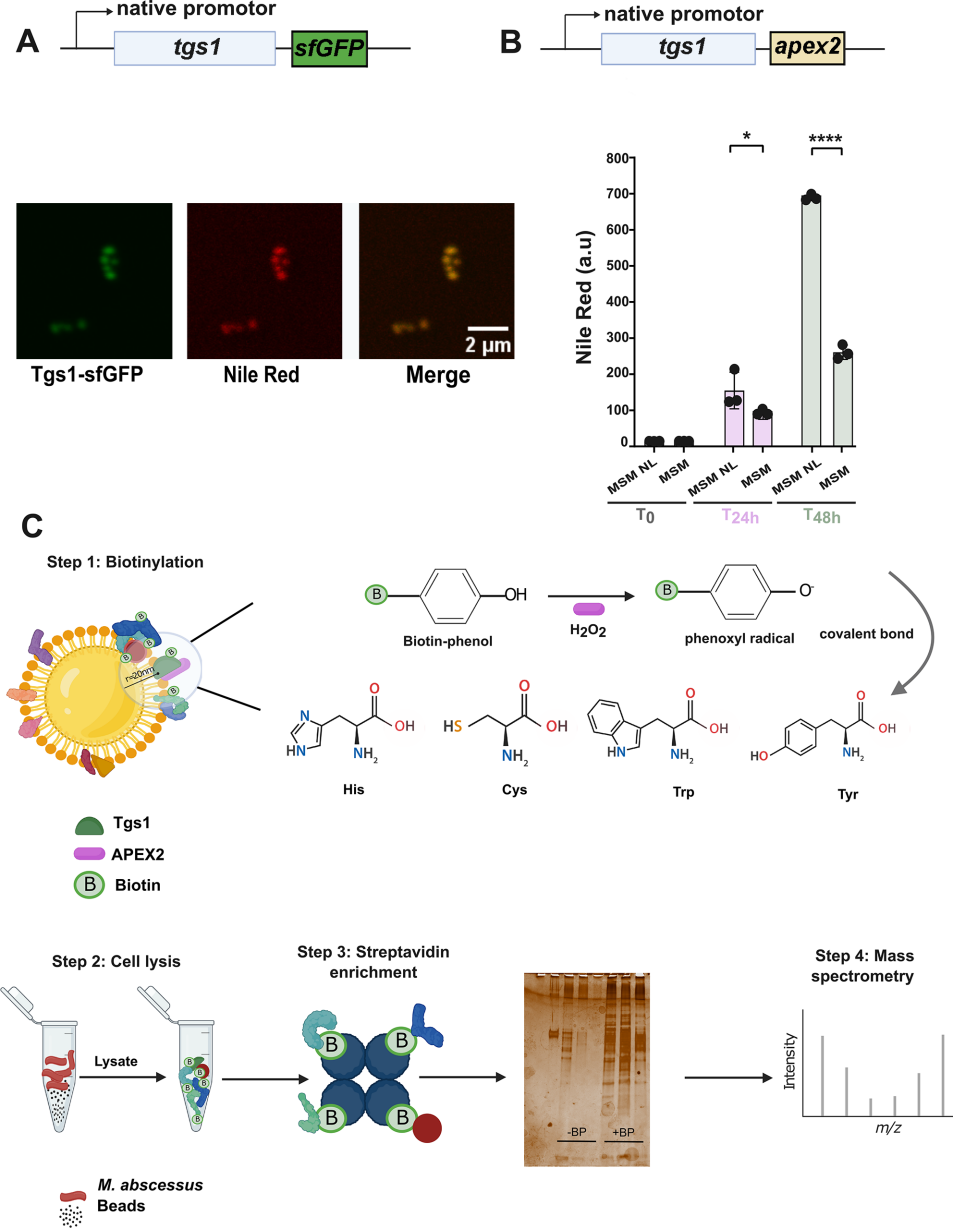
Setting up the APEX2 system. (**A**) Diagram of the construct encoding tgs1 fused with *sf*GFP under the endogenous promoter. *Mab* cells were grown in MM NL and fixed in paraformaldehyde. Fluorescence microscopy images showing Nile red (559/635 nm), *sf*GFP (488/510 nm), and merge channels of cells containing ILI colocalizing with Tgs1. (**B**) Diagram illustrating the construct encoding the native promoter and full genomic sequence of tgs1 fused with apex2 via a linker domain. Data represent the mean of Nile Red fluorescence accumulation in three biological replicates measured by flow cytometry. Statistical analyses were carried out using a two-way ANOVA with a Šidák test. * *P*-value < 0.05, ** *P*-value < 0.01, *** *P*-value < 0.001, and **** *P*-value < 0.0001. (**C**) Representative workflow depicting APEX2 action with the chosen bait (Tgs1) in green and APEX2 molecule in purple. The main amino acids interacting with biotin-phenol (BP) are shown by their chemical formula. Silver staining of SDS-PAGE gel: a representative silver-stained SDS-PAGE gel of treated (+BP) and untreated (−BP) lysate following streptavidin enrichment, at 24 and 48 h, respectively. Thirty-five nanogram of BSA was loaded as a control. Images were taken using the Chemidoc MP imaging system (Bio-Rad). Color information was preserved to display the different hues produced by the stain. No other digital manipulation was performed.

To specifically identify IAP, we adapted the APEX2 approach in an *in vitro* lipid accumulation model (3, 21). However, it was crucial to use bait at the ILI surface contributing to TAG synthesis, given APEX2’s cytoplasmic localization (27). To confirm Tgs1 as a genuine IAP in *Mab*, as suggested in *Mbv* BCG (11), the protein was fused with a superfolder green fluorescent protein (*sf*GFP)-tag to study ILI colocalization (Fig. 2A). Western blotting confirmed Tgs1 expression at both 24 and 48 h during the synthesis phase (Fig. S1). Therefore, APEX2 was directed to ILI via an integrative plasmid containing *tgs1* with its own promoter fused with the *apex2* coding sequence via a linker domain (Fig. 2B). Nile Red signal quantification by flow cytometry revealed higher fluorescence levels in MSM NL at 48 h (700 a.u) compared to 24 h (150 a.u) or MSM at 48 h (270 a.u). Moreover, the complementation of the Δ*tgs1* mutant strain with pMV306-p*tgs1-apex2* restored the wild-type (WT) TAG content, confirming the functionality of the Tgs1-APEX2 fusion protein in *Mab* (Fig. S2).

In the presence of biotin phenol, APEX2 biotinylates proteins within a 20 nm radius by generating short-lived phenoxyl radicals (1 ms; Fig. 2C) (31). To avoid contamination with endogenous biotinylated proteins, these latter, identified in the control group (MSM NL without biotin phenol), were removed from the analysis. Silver staining was performed after streptavidin enrichment for all lysates, both with and without BP, to confirm that ILI-targeted APEX2 biotinylates proteins exclusively under conditions of biotin phenol addition.

Recombinant *Mab*-producing Tgs1-APEX2 was grown in MSM NL and pulsed with biotin phenol and hydrogen peroxide. From the bacterial lysates, biotinylated products were enriched using streptavidin-coated beads, followed by protein stripping, trypsin digestion, and LC-MS/MS analysis. This assay identified 228 highly enriched proteins, with 85 ones present at 24 h, 105 proteins at 48 h, and 38 at both time points, establishing APEX2-mediated protein tagging as a suitable method for localizing proteins to the ILI surface. However, some limitations are inherent to this technique. First, target proteins should possess tyrosine, cysteine, histidine, or tryptophan residues on their surface for efficient biotinylation by biotin phenol. Second, *Mab* possesses endogenous biotinylated proteins, such as pyruvate carboxylase MAB_3267c and acetyl-CoA/propionyl-CoA carboxylase MAB_1876c (32), excluded from our analysis but that could, eventually, be involved in ILI synthesis. Overall, the use of APEX2 seems appropriate for probing cytoplasmic protein-protein interactions, consistent with established methodologies for examining eukaryotic lipid droplet proteomes (26, 27).

### Deciphering the ILIome using the Tgs1-APEX2 strategy

The enriched biotinylated proteins derived from the *Mab* strain producing Tgs1-APEX2 were analyzed by MS. Proteins identified at 24 and 48 h are listed in Tables S3 and S4.

**Fig 3:**
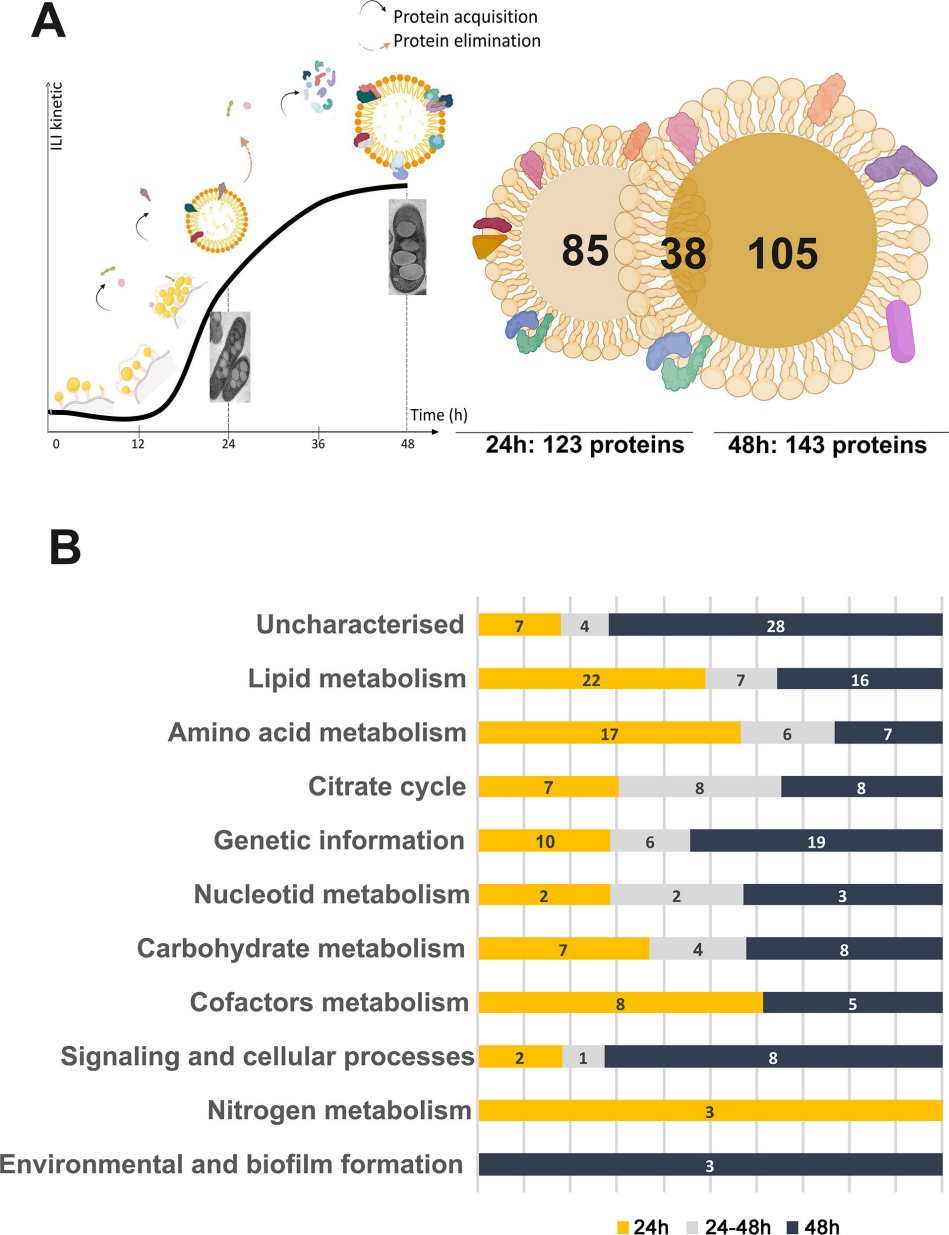
Target selection by APEX2. (**A**) ILI biosynthesis kinetics adapted from Dargham et al. (33), followed by a Venn diagram representing selected targets at 24 h and 48 h based on a −Log(*P*-value) threshold of 1.3 and a fold change (Log_2_) of 1. Thin sections of *in vitro* cultures of *Mab* in MSM NL at 24 and 48 h, respectively. (**B**) Distribution of the selected IAP candidates based on their respective KEGG pathways (https://www.genome.jp/kegg/) at each time point. Yellow bars represent 24 h, blue bars represent 48 h, and gray bars represent 24–48 h.

Due to the sensitivity of the method, at 24 h in MSM NL, 463 proteins were identified. However, by applying a *P*-value ≤ 0.05 (i.e., -Log [*P*-value] > 1.3) and a fold change (Log_2_) ≥1, this set was reduced to 123 proteins significantly more abundant in MSM NL compared to MSM. At 48 h, 624 proteins were identified, with 143 meeting the cutoff criteria. Notably, 38 were present at both time points, resulting in a set of 228 distinct proteins enriched at both time points compared to the uninduced control, highlighting the dynamic nature of ILI synthesis (33, 34). Differences in ILI characteristics, such as size, composition, hydrophobicity, and surface tension, between the 24 and 48 h time points suggest that certain proteins may have varying affinities for distinct lipid molecules or, as already proposed (35, 36), require specific structures like anchors, beta hairpins, or amphipathic helices to efficiently interact with ILI (Fig. 3A). It has been suggested that in prokaryotes, these amphipathic helices are commonly involved in interactions, considering that these structural motifs as essential for understanding how proteins grasp with lipid droplets, particularly in prokaryotic systems (14, 20).

Some proteins (7/85 at 24 h, 28/105 at 48 h, and 4/38 at both 24 and 48 h) have unknown predicted functions (Fig. 3B). Proteins associated with lipid metabolism were equally represented in abundance at 24 h (22/85) and 48 h (16/105), while those engaged in amino-acid and carbohydrate metabolism represented 24/85, 17/105, and 10/38, respectively, of the 228 distinct proteins (Fig. 3B; Table S5). These findings align with previous *in silico* analyses (20), where only 17% of identified proteins were classified in the lipid metabolism functional category. These data support the emerging understanding of the physiological function of ILI, primarily designed for lipid storage but also crucial for cellular homeostasis under challenging conditions (6, 37).

From the pool of 45 protein candidates annotated in the lipid metabolism category, 10 were chosen for subsequent validation as true IAP. This first set of proteins was selected based on the following criteria: they are encoded by non-essential genes and share orthologs in *Mtb* with ≥25% sequence identity (Table 1). Genes encoding transcription factors were excluded from this selection.

**TABLE 1:** Selected IAPs after 24 h and 48 h culture of *Mab* in MSM NL using Tgs1-APEX2 as bait and 558 identified by LC-ESI-MS/MS analysis by applying *P*-value < 0.05 and Fold change (Log2) > 1 threshold values^*a*^

24 h	48 h	Genes	Protein names			*Mtb* orthologs	
				Rv number	Sequence identity (%)	Essentiality	Functional category
+^*b*^		*MAB_3040* c	Probable acyl-CoA dehydrogenase	Rv2724c	77.3	Non-essential	Lipid metabolism
+		*MAB_1187* c	Probable enoyl-CoA hydratase	Rv1071c	70.5	Non-essential	Lipid metabolism
+		*MAB_4437*	Probable acyl-CoA dehydrogenase FadE5	Rv0244c	79.1	Non-essential	Lipid metabolism
+	+	*MAB_2737*c	Probable enoyl-CoA hydratase/isomerase	Rv1472	75.7	Non-essential	Lipid metabolism
+	+	*MAB_4532*c	Uncharacterized N-acetyltransferase	Rv2416c	28.7	Non-essential	Virulence, detoxification, and adaptation
	+	*MAB_1463*	Probable acetyl-CoA acetyltransferase FadA4	Rv1323	76.5	Non-essential	Lipid metabolism
	+	*MAB_3481*	Probable acyl-CoA dehydrogenase FadE4	Rv0231	52.9	Growth-advantage	Lipid metabolism
	+	*MAB_3486*	Probable acyl-CoA dehydrogenase	Rv3139	69.4	Non-essential	Lipid metabolism
	+	*MAB_1917*	Diacylglycerol O-acyltransferase	Rv1760	56.2	Non-essential	Lipid metabolism
	+	*MAB_2806*	Lipoprotein LprG	Rv1411c	42.2	Non-essential	Cell wall and cell processes

^*a*^ *Mtb* orthologs were retrieved using the KEGG database (38, 39) and then cross-referenced with the Mycobrowser (40) database for accurate Protein names, Rv numbers, and sequence identity (%). The Essentiality and the Functional category related to *Mtb* orthologs were from Dejesus et al. and Camus et al., respectively (41, 42).

^*b*^ "+"means the presence of protein at 24 h or 48 h.

Among the selected protein candidates, four acyl-CoA dehydrogenases (MAB_4437, MAB_3040, MAB_3481, and MAB_3486) were identified at 24 h and 48 h, along with Tgs2 (MAB_1917; detected at 48 h) and an acyl-CoA acetyltransferase (MAB_1463). A probable enoyl-CoA hydratase (MAB_1187c) was also identified at 24 h. Two proteins with diverse functions (MAB_4532c and MAB_2737c) were common at both time points (Fig. 3A). The lipoprotein LprG (MAB_2806) was identified at 48 h and selected based on its role in TAG transport (43). LprG being located in the inner membrane (43), its identification with APEX2 was expected. However, at 48 h, when the ILI size is optimal, APEX2 may biotinylate LprG due to close contact between the mature ILI and the inner membrane.

To assess the potential presence of amphipathic helices in our selected candidate targets (14, 20), we employed Heliquest software (http://heliquest.ipmc.cnrs.fr). Remarkably, 7 out of 10 candidates exhibited at least one amphipathic helix, utilizing the same cut-off criteria as in our previous studies (Fig. S3) (14, 20). MAB_4532c, MAB_2806, and MAB_1917 did not show apparent amphipathic helix structures. Therefore, alternative structural patterns may play a role in the interaction between these proteins and ILI.

### What are the impacts of these selected targets on ILI synthesis?

**Fig 4:**
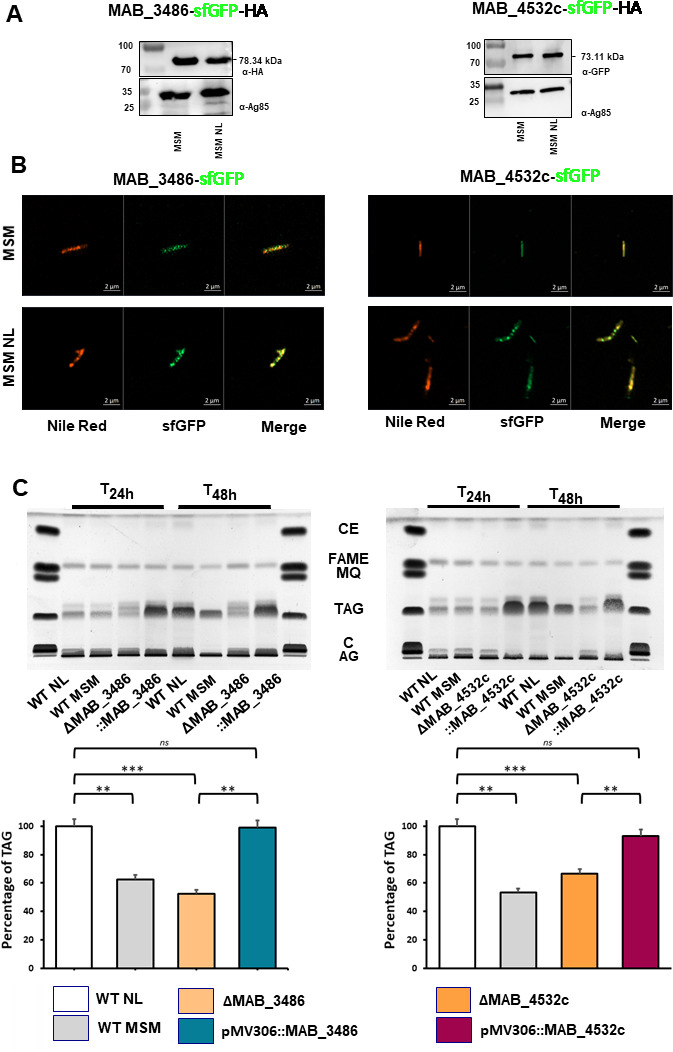
Validation of IAP mutants in *Mab*. (**A**) *Mab* was transformed with plasmids allowing overexpression of the genes of interest fused with sfGFP for colocalization studies. The western blots show the fusion between the selected proteins and sfGFP (28 kDa). Ag85 was included as a loading control. (**B**) Bacteria were grown in MSM and MSM NL for 48 h, fixed with 2% paraformaldehyde, stained with Nile Red, and visualized by fluorescence microscopy. Scale bars, 2 µm. (**C**) Deletion mutants and complemented Mab strains were grown in MSM NL, and cultures were collected at 24 h and 48 h. After lyophilization, equal amounts of dry cells were used for apolar lipid extraction. TAG levels from each culture were analyzed by thin-layer chromatography (TLC). Each TLC plate is representative of experiments performed in triplicate. Densitometry results for 48 h are expressed as mean values ± SD of three biologically independent experiments and reported as the percentage of TAG levels relative to those in *Mab* WT grown in MSM-NL (set at 100%). Statistical significance was assessed with one-way ANOVA followed by Tukey’s multiple comparisons posthoc test using Prism 8.0 (GraphPad, Inc): ** *P*-value < 0.01; *** *P*-value < 0.001; ns, not significant (*P*-value > 0.05). C: cholesterol; CE: cholesterol ester; FA: fatty acid; FAME: fatty acid methyl esters; MQ: mena quinone.

Since *sf*GFP alone does not colocalize with ILI (Fig. S4), the 10 chosen IAP gene candidates were then fused with *sf*GFP and overexpressed in *Mab* under each gene’s native promoter to confirm their intracellular localization (Fig. 4A; Fig. S1 and S5). In MSM, a diffuse fluorescence signal was observed, suggesting a lipid-poor bacterium with a homogeneous protein distribution in the cytoplasm (Fig. 4B). Conversely, in MSM NL, well-defined regions marked by Nile Red indicated ILI-positive bacteria with clear colocalization with the *sf*GFP signal (Fig. 4B; Fig. S5). Eight fusion proteins successfully colocalized with Nile Red (MAB_3481, MAB_3486, MAB_1917, MAB_4437, MAB_1187, MAB_2737c, MAB_4532c, and MAB_3040), validating the effectiveness and selectivity of APEX2 technology to identify IAP. Western blot analysis using anti-*sf*GFP or anti-HA antibodies showed equal protein production in MSM and MSM NL. Thus, the co-localization of the proteins with ILI is not related to increased expression levels in MSM NL but relies on protein redistribution to the ILI surface. Unfortunately, MAB_2806 and MAB_1463 were not produced at detectable levels by western blotting (data not shown), thus preventing localization analyses, and were not further studied.

To assess the impact of each designated IAP on ILI formation, double homologous recombination was employed, as described previously (44), to delete the eight corresponding genes (*MAB_2737c*, *MAB_1917*, *MAB_1187c*, *MAB_4532c*, *MAB_3040c*, *MAB_4437*, *MAB_3486*, and *MAB_3481*) in *Mab* (Fig. S6).

After verifying proper gene deletion by PCR amplification and sequencing (Fig. S7) and bacterial growth in all mutants (Fig. S8), TAG accumulation was subsequently examined by thin-layer chromatography (TLC; Fig. S9A). We reasoned that a defect in TAG accumulation at 24 h would affect the final TAG quantity at 48 h. TAG production was quantified at 48 h only, avoiding difficulty in obtaining enough bacteria at 24 h for lipid analysis. Three phenotypes were observed at 48 h (Fig. S9A). Deletion of either *tgs2* (*MAB_1917*) or the acyl-CoA dehydrogenase (*MAB_4437*) in *Mab* showed no impact on TAG accumulation as compared to the WT strain. These data are not surprising, as the *tgs2* gene has been shown to have a minimal impact on ILI synthesis in *Mab* (8). Furthermore, the *Mab* genome contains a large number of other genes exhibiting similar activity, suggesting that other redundant proteins may compensate for the absence of *tgs2*. The deletion of other genes showing the second kind of phenotype (two acyl-CoA dehydrogenases *MAB_3040* and *MAB_3481* and two enoyl-CoA hydratases *MAB_1187* and *MAB_2737* c) resulted in a modest (25%–40%) reduction in TAG levels (Fig. S9), suggesting an indirect or secondary role in TAG accumulation. The *Mtb* ortholog of MAB_2737 c (Rv1472/EchA12), involved in fatty acid oxidation, may play a role in phosphatidyl-*myo*-inositol mannoside elongation (45). We propose that these IAPs may have an impact on membrane lipids as well as TAG accumulation. However, further studies and biochemical characterization are needed to better understand their roles and localization on the ILI. The third observed phenotype is linked to *MAB_*3486 (corresponding protein identified at 48 h; Table 1) and *MAB_4532* c (corresponding protein identified at 24 and 48 h), whose deletion mutants displayed significant reductions (57% and 49%) in TAG levels, respectively (Fig. 4C). Complementation of the mutants with an integrative vector (Fig. S10) restored the WT TAG levels (Fig. 4C), confirming the primary role of MAB_3486 and MAB_4532c in TAG biosynthesis, despite not being in the core ILIome reported earlier (20). Analyzing the acylglycerol content in Δ*MAB_3486* and Δ*MAB_4532*c revealed higher amounts of free fatty acids compared to the WT strain, which disappeared after complementation (Fig. S11). This strengthens the idea that while both mutants have limited TAG production, they accumulate free fatty acids, which are TAG biosynthetic intermediates used by the WT and complemented strains to generate their TAG pools.

The ortholog of MAB_3486 in *Mtb* is FadE24 (Rv3139), belonging to the FadE23-FadE24 complex, repressed by cholesterol and involved in fatty acid recycling, particularly during β-oxidation (46, 47). This may explain the important reduction of TAG formation in Δ*MAB_3486* following β-oxidation inhibition. However, the complex has a different catalytic activity than dehydrogenating the acyl-CoA and is upregulated in the presence of isoniazid (48). Indeed, FadE23-FadE24 is controlled by *SigE*, which helps manage environmental stresses. Therefore, its presence in our biological model, which is based on nutrient stress, aligns with these findings.

The structure of MAB_4532c, also known as Eis2 in *Mab*, belongs to the GNAT (GCN5-related N-acetyltransferase) family (49). Knocking out this gene in *Mab* reduces bacterial survival within murine macrophages (50). Notably, Eis2 is known to confer resistance to aminoglycoside antibiotics, and numerous inhibitors targeting this protein have been developed, emphasizing its potential significance as a therapeutic target against mycobacteria (51). Although Eis2 acetylates the primary amine of aminoglycosides (49), the endogenous substrate of this enzyme in mycobacteria remains to be established. Thus, the specific mechanism by which Eis2 interacts with ILI remains elusive and has yet to be determined.

At 48 h, two major acyltransferases, PlsB (MAB_1984) and PlsM (MAB_0165), were identified, as highlighted by Angala et al. (52). These two enzymes, one of which is essential, are involved in phosphatidic acid biosynthesis leading to TAG accumulation. However, they were excluded from our study because they did not meet the initial selection criteria for further characterization (MAB_1984 FC 1.24, Log_10_[*P*-value] = 2.04, and MAB_0165 FC 1.25, Log_10_[*P*-value] = 1.81), but their presence underscores their potential importance, warranting future investigation to elucidate their specific roles in ILI formation.

### Conclusion

ILIs represent a fascinating aspect of bacterial physiology (5, 33). These lipid-loaded organelles are formed within the bacterial cytoplasm through the accumulation of TAG and derived from lipid-rich infected cells or from *de novo* biosynthesis of TAG. Besides energy storage, it has been clearly established that lipid metabolism in actinomycetes—particularly ILI formation and degradation—plays a crucial role in survival, persistence, pathogenicity, and drug tolerance.

Lipid droplet-associated proteins encompass various families such as structural proteins, signaling proteins, membrane trafficking components, metabolic enzymes, and proteins implicated in protein degradation and binding to DNA (53–55). Although IAP has not been extensively investigated, their significance is progressively gaining elucidation. Increasing evidence points to the pivotal role of mycobacterial ILI in virulence. Though studies on *Msmeg* and *Mbv* BCG have explored IAP (11, 14), direct identification and validation of IAP remain largely incomplete. Identifying and characterizing these interacting enzymes would enhance our understanding of ILI metabolism in pathogenic mycobacteria, offering new approaches for managing mycobacterial-related diseases.

This study demonstrates the use of APEX2 for the first time on prokaryotic lipid droplets and validated the technology for identifying new IAP. Through proximity labeling in a nutrient/nitrogen-starved model, we identified 228 proteins potentially involved in a direct or indirect role in ILI biogenesis. This complex process is highly dynamic, involving numerous protein families with overlapping activities. Based on TAG quantification, MAB_3486 and MAB_4532c were found to be the most important proteins among the 10 selected candidates. Overall, this is the first comprehensive study on mycobacterial ILI, providing a detailed proteomic view of ILI formation. APEX2 paves the way for a complete ILIome, mapping proteins involved in ILI degradation. Combining APEX2 with TurboID (56), known as TransitID, could help track a specific proteome from the beginning of biogenesis to ILI maturation.

Although more work is needed to validate additional IAP, this work enhances our understanding of mycobacterial lipid metabolism. In the long term, it may help discover new therapeutic targets for future treatments against *Mab* diseases.

## MATERIALS AND METHODS

### Bacterial strains and growth conditions

*Escherichia coli* DH10B cells used for cloning were cultured on Luria-Bertani agar plates at 37°C, supplemented with 50 µg/mL kanamycin when necessary.

The smooth (S) variant of *Mab* CIP104536^T^ was cultivated in Middlebrook 7H9 liquid medium (BD Difco, Le Pont de Claix, France) supplemented with 0.05% Tween 80 (Sigma-Aldrich, Saint-Quentin Fallavier, France), 0.2% glycerol (Euromedex, France), and 10% oleic albumin dextrose catalase (OADC enrichment, BD Difco, France; 7H9-S^OADC^) at 37°C with shaking at 200 rpm. ILI accumulation in *Mab* liquid culture was performed as previously described with slight modifications (3) (refer to supplementary data for details).

### Plasmid construction and cloning

Primers used in this study are detailed in Table S6 and were synthesized by Integrated DNA Technologies. The *MAB_3551*c (*tgs1*) gene was PCR amplified using Phusion High-Fidelity Enzyme (ThermoFisher Scientific, Waltham, MA), fused with *apex* or *gfp* gene, and cloned using the SLIC method (57) into the pMV306 containing a kanamycin resistance cassette under the endogenous *tgs1* promoter. The sequence of each clone was validated by GATC Eurofins (Germany).

The deletion of each selected gene from the *Mab* genome was performed as previously described (44) (Fig. S5). Briefly, electrocompetent cells were transformed with pUX1-*katG-target*. Red kanamycin-resistant colonies underwent a second selection based on isoniazid resistance and loss of red fluorescence. Screening by PCR and sequencing validated each deletion mutant strain (Fig. S6; Table S6) (58).

### Bacterial fixation and Nile Red staining

Bacterial cells cultured in MSM or MSM NL were harvested by centrifugation at 3,500 g for 10 min at 20°C, fixed with 2% paraformaldehyde in PBS for 1 h at room temperature, washed twice with PBS-Tween 80 (0.05%, [vol/vol]), re-suspended in PBS to a theoretical OD_600 nm_ of 10, and incubated with Nile Red fluorescent dye (Interchim, Montluçon, France) for ILI staining, as described (3). The bacterial suspension was visualized using an Olympus IX81 confocal microscope equipped with a UPlanSApo 100 × 1.40 NA objective and operated with the FV1000 software.

### Flow cytometry

Bacterial populations studied by flow cytometry were at OD_600 nm_ = 0.1. Fixed cells were stained with Nile Red. Scatter plots obtained (forward scattering FSC vs side scattering SSC signals) were gated on the population of interest, filtered to remove multiple events, and then analyzed for fluorescence intensity (FL3 615/25 nm) using both 488 and 561 nm lasers excitation. For each biological sample, 300,000 events were measured. Data were acquired with an S3E cells sorter (BioRad) and analyzed and plotted using FlowJo v10.8. Data represent at least three biological replicates, and statistical analyses were performed with Prism v8.2 software using two-way ANOVA.

### Whole-cell protein extract

Bacteria were centrifuged, washed twice in PBS, and re-suspended in PBS-urea 8 M (Euromedex) at pH 8, then sonicated for complete lysis. The lysate was recovered after centrifugation at 4°C for 5 min at 1,300 g, and protein concentration in the supernatant was determined using the Bradford method (Bio-Rad). SDS-PAGE was performed after loading 100 µg of lysate, while 50 µg was used for mass spectrometry.

### Biotinylation reaction

*Mab* harboring the pMV306-*tgs1-apex2* was grown in MSM and MSM NL at 37°C under agitation at 200 rpm (3). Cells were harvested by centrifugation for 10 min at 3,500 g at 20°C, washed twice with PBS, and re-suspended in PBS to an OD_600 nm_ of 40. Biotin phenol (100 mM, Iris Biotech) was added, followed by incubation at 37°C, 200 rpm, in the dark for 30 min. Hydrogen peroxide (100 mM) was then added under the same conditions for exactly 2 min. The reaction was stopped by adding 1 mL of 2× quenching solution composed of PBS, 1 M sodium ascorbate (Sigma), and 1 M sodium azide (Sigma). After centrifugation at 3,500 g for 10 min at 20°C, pellets were washed twice with 1 mL of 1× quenching solution and 1 mL of PBS-Tween 80 (0.05%, [vol/vol]) each, resuspended in 2 mL ice-cold PBS-protease inhibitor cocktail (c*O*mplete Mini, EDTA-free, Sigma-Aldrich) (22), and lysed using a Mini-Beadbeater96 (BioSpec) for 3 × 4 min with 250 µL of glass beads (0.1 mm-diameter glass beads, BioSpec). The lysates were centrifuged for 5 min at 1,300 g at 4°C, and the supernatant was collected to determine the protein concentration.

### Streptavidin enrichment

Twenty microliter of 10 mg/mL streptavidin agarose resin (ThermoFisher Scientific, Waltham, MA) was washed twice with 500 µL of PBS and centrifuged for 5 min at 5,000 g. In parallel, 1 µg of each lysate was heated at 95°C for 5 min. Streptavidin was then mixed with the heated lysates and incubated under gentle rotation (15 rpm) for 3 h. Beads were washed with 200 µL PBS-20% SDS and incubated for 20 min on the orbital shaker (15 rpm/min) at room temperature. After two PBS washes and centrifugation at 5,000 g for 5 min, SDS loading dye 1 × was added, and the sample was boiled for 10 min at 95°C.

### Mass spectrometry

1. The whole proteome analysis is detailed in the supplementary data. Briefly, proteins were first stacked as a single band using an acrylamide gel. After in-gel digestion, extracted peptides were analyzed by LC-MS/MS using an Orbitrap Fusion Lumos Tribrid Mass Spectrometer (ThermoFisher Scientific, San Jose, CA). Label-free quantification (LFQ) was processed using the DIA-NN 1.8 algorithm using the *Mab* database (UP000007137) extracted from UniProt on 10 March 2021, which contains 4,940 entries. The DIA-NN primary output file was filtered at 1% FDR, and LFQ intensity was recalculated using the DIAgui package at 1% *q*-value (59). Statistical analysis was performed using the Perseus program (version 1.6.15.0) from the MaxQuant environment (http://www.maxquant.org). To determine whether a given detected protein was specifically differential, a two-sample *t*-test was performed using permutation-based FDR control at 0.05.
2. For the biotinylated proteome, protein samples were prepared similarly, and analysis was conducted using a Q Exactive Plus Hybrid Quadrupole-Orbitrap (ThermoFisher Scientific, San Jose, CA) in data-dependent acquisition mode. Relative intensity-based LFQ was processed using the MaxLFQ algorithm available on the MaxQuant computational proteomics platform, version 1.6.3.4. Statistical analysis was performed similarly to the whole proteome analysis.

The mass spectrometry proteomics data have been deposited to the ProteomeXchange Consortium (www.proteomexchange.org) (60) via the PRIDE partner repository (61) with data sets identifiers PXD052312 (Reviewer account details: username: reviewer_pxd052312@ebi.ac.uk password: QbTFUBeniAp1) and PXD052310 (Reviewer account details: username: reviewer_pxd052310@ebi.ac.uk password: wmd2IwZGGO98).

### Lipid extraction and TLC analysis

Mycobacteria grown in MSM NL until reaching an OD_600 nm_ of 1 were collected after 24 or 48 h and processed for apolar lipids extraction and quantification by TLC (3) (see Supplementary data for details).

## SUPPLEMENTAL MATERIAL

Supplemental Material mbio.01475-24-s0001.docx Supplemental methods and figures.

Supplemental Tables mbio.01475-24-s0002.xlsx Tables S1 to S6.

ASM does not own the copyrights to Supplemental Material that may be linked to, or accessed through, an article. The authors have granted ASM a non-exclusive, world-wide license to publish the Supplemental Material files. Please contact the corresponding author directly for reuse.
