# Supplementary material for: A proteomic and functional view of intrabacterial lipid inclusion biogenesis in mycobacteria
Source: mBio. 2025 Feb 25;16(4):e01475-24. doi: 10.1128/mbio.01475-24 (PMC11980559; doi:10.1128/mbio.01475-24)
Supplement: Supplemental Material — Supplemental methods and figures. [file mbio.01475-24-s0001.docx]

**SUPPLEMENTARY DATA**

**A proteomic and functional view of intrabacterial lipid inclusion biogenesis in mycobacteria**

Tonia Dargham^1,2,^*, John Jairo Aguilera-Correa^3,^*, Romain Avellan^1,^*, Ivy Mallick^1^, Léa Celik^1^, Pierre Santucci^1^, Gael Brasseur^4^, Isabelle Poncin^1^, Vanessa Point^1^, Stéphane Audebert^5^, Luc Camoin^5^, Wassim Daher^3,6^, Jean-François Cavalier^1^, Laurent Kremer^3,6^ & Stéphane Canaan^1§^

^1^ Aix-Marseille Univ., CNRS, LISM UMR 7255, IMM FR3479, IM2B, Marseille, France

^2^ IHU Méditerranée Infection, Aix-Marseille Univ., Marseille, France

^3^Centre National de la Recherche Scientifique UMR 9004, Institut de Recherche en Infectiologie de Montpellier (IRIM), Université de Montpellier, 1919 route de Mende, 34293, Montpellier, France.

^4^ Aix-Marseille Univ., CNRS, LCB UMR 7283, IMM FR3479, IM2B, Marseille, France

^5^ Aix-Marseille Univ., INSERM, CNRS, Institut Paoli-Calmettes, CRCM, Marseille, France

^6^INSERM, IRIM, 34293 Montpellier, France.

* Authors contributed equally to this work, and should be considered as first co-authors

^§^ Correspondence address to Stéphane Canaan, canaan@imm.cnrs.fr.

**Content**

**Supplementary methods**

**Supplementary figures**

**Figure S1:** Validation of the sfGFP fusion to each selected protein.

**Figure S2:** Comparison of Nile Red intensity staining in *M. abscessus* carrying pMV306-*tgs1-apex2* and WT strains.

**Figure S3:** Detection of an amphipathic helix in each selected protein**.**

**Figure S4: sf-GFP does not localize with ILI when assessed during nitrogen limiting-condition.**

**Figure S5:** Colocalization of each selected IAP with ILI.

**Figure S6**: Unlabeled deletion strategy for the deletion of *MAB_3486* in *M. abscessus*.

**Figure S7:** Genetic validation of each *M. abscessus* deletion mutant.

**Figure S8:** Growth curves for the deletion mutants**.**

**Figure S9:** Apolar lipid profile in IAP deletion genes.

**Figure S10: Validation of the sfGFP-tagged complementation of each *M. abscessus* mutant.**

**Figure S11: Acylglycerol migration profile** in IAP deleted mutants.

**Supplementary methods**

ILI accumulation in *M. abscessus*

Briefly, from a logarithmic growth phase culture (OD_600_ ~1-1.5), *M abscessus* cells were harvested by centrifugation at 3,500*g* for 10 min. Pellets were washed twice with sterile phosphate-buffered saline (PBS) (pH 7.4) containing 0.05% (*v/v*) Tween-20, then once with PBS, and finally re-suspended in PBS at a final theoretical OD_600_ of 10. This bacterial suspension was used to inoculate at an initial OD_600_ of 0.1, either fresh Mineral Salt Medium (MSM) (2 g/L Na_2_HPO_4_, 1 g/L KH_2_PO_4_, 0.5 g/L NaCl, 0.2 g/L MgSO_4_, 20 mg/L CaCl_2_, 1 g/L NH_4_Cl, and 5% glycerol) or Mineral Salt Medium Nitrogen Limited (MSM NL) containing only 0.15 g/L NH_4_Cl. To prevent bacterial clumping, Tyloxapol (Sigma-Aldrich) was added at a final concentration of 0.02% (*v*/*v*). The MSM and MSM NL culture media were then incubated for 24 and 48h at 37°C under shaking (200 rpm).

*SDS PAGE and Western blotting*

After 24h or 48h of culture in MSM or MSM NL, bacterial pellets were recovered, and total lysates were denatured by incubating with 1×SDS loading dye for 10 min at 95 °C and subjected to SDS-PAGE. Proteins were transferred onto 0.2 µm polyvinylidene difluoride (PVDF) using the Trans-Blot Turbo transfer system at 25 V, 1 A for 7 min (Bio-Rad, Hercules, CA). The membrane was then blocked in TBS-T (0.3% BSA) and incubated with a primary antibody anti-APEX2 (1 µg/ml, Thermo Fisher Scientific, Waltham, MA) overnight at 4 °C. After three quick washes with TBST for 10 min each, the membrane was incubated for 1 h with the secondary anti-llama antibody. Detection was achieved using the Pierce ECL Western blotting substrate solution (Thermo Fisher Scientific, Waltham, MA) and visualized using the ChemiDoc MP imaging system (Bio-Rad).

Bacteria were harvested, resuspended in PBS, and disrupted by bead beating using 1 mm diameter glass beads. Protein concentration was assessed using the BCA Protein Assay Reagent kit, according to the manufacturer’s instructions. Equal amounts of proteins (50 mg) were separated by SDS-PAGE and transferred to a nitrocellulose membrane. For GFP detection, nitrocellulose membranes were probed for 1h with mouse anti-GFP antibodies (dilution 1:2,000). Anti-GFP antibodies were diluted in 5% non-fat milk powder in TNT buffer (50 mM Tris pH 8; 150 mM NaCl; and 0.05% Tween 20). After washing, membranes were incubated for 45 min with goat anti-mouse antibodies conjugated to HRP (dilution 1:5,000). The GFP signal was revealed using a ChemiDoc MP system for imaging and analyzing gels.

*1-Whole proteome analysis:* For relative proteomic analysis, 15 µg of proteins were solubilized in LDS sample buffer (2× concentrated) (Invitrogen, Life Technologies), before loading on NuPAGE™ 4–12% Bis–tris acrylamide gels according to the manufacturer’s instructions (Invitrogen, Life Technologies). Running of samples was stopped as soon as proteins stacked as a single band, and following imperial blue staining (Life Technologies), the upper part of the gel containing the proteins was cut and processed for classical in-gel digestion (washes, thiols reduction with 10 mM DTT, and cysteine alkylation with 55 mM iodoacetamide). Extracted peptides were concentrated under speed-vacuum. Samples were reconstituted with 0.1% trifluoroacetic acid in 2% acetonitrile and analyzed by liquid chromatography (LC)-tandem MS (MS/MS) using an Orbitrap Fusion Lumos Tribrid Mass Spectrometer (ThermoFisher Scientific, San Jose, CA) online with a nanoRSLC Ultimate 3000 chromatography system (ThermoFisher Scientific, Sunnyvale, CA). First, peptides were concentrated and purified on a pre-column PepMap100 C18, 2 cm × 100 µm I.D, 100 Å pore size, 5-µm particle size in solvent A (0.1% formic acid in 2% acetonitrile). In the second step, peptides were separated on a reverse phase LC EASY-Spray C18 column PepMap RSLC C18, 50cm $\times$ 75 µm I.D, 100 Å pore size, 2-µm particle size (ThermoFisher) at 300 nL/min flow rate and 40°C. After column equilibration using 4% of solvent B (20% water - 80% acetonitrile - 0.1% formic acid), peptides were eluted from the analytical column by a two-step linear gradient (2-20% acetonitrile/H_2_O; 0.1% formic acid for 90 min and 20-45% acetonitrile/H_2_O; 0.1% formic acid for 20 min). For peptide ionization in the EASY-Spray nanosource in front of mass spectrometer, spray voltage was set at 2.2 kV and the capillary temperature at 275°C. The Orbitrap Lumos was used in data-independent mode with the following parameters. First, MS spectra were acquired in the Orbitrap in the range of m/z 375-1,500 at a FWHM resolution of 120,000 measured at 400 m/z. AGC target was set at standard parameters with an automatic Maximum Injection Time. MS2 spectra were acquired in the Orbitrap with a resolution of 30,000, in the mass range of 200-1,800 m/z after isolation of parent ion in the quadrupole and fragmentation in the HCD cell under collision Energy of 30%. DIA parent ion range was from 400 to 1,000 m/z divided into 40 windows 16 Da wide and from 1,000 to 1,500 m/z divided into 10 windows 50 Da wide.

Relative intensity-based label-free quantification (LFQ) was processed using the DIA-NN 1.8 algorithm. Spectra were searched against the *M. abcessus* database (UP000007137) extracted from UniProt on March 10, 2021, containing 4940 entries, with the addition of a protein contaminant bank (1). The search parameters included: *(i)* trypsin allowing cleavage before proline; *(ii)* one missed cleavages were allowed; *(iii)* cysteine carbamidomethylation (+57.02146) as a fixed modification, and methionine oxidation (+15.99491) and N-terminal acetylation (+42.0106) as variable modifications; *(iv)* a maximum of 1 variable modification per peptide was allowed; and *(v)* a minimum peptide length was 7 amino acids and a maximum of 30 amino acids. The match between runs option was enabled to transfer identifications across different LC-MS/MS replicates based on their masses and retention time. The precursor false discovery rate was set to 1%. DIA-NN parameters were configured on Single-pass mode for Neural Network classifier, Robust LC High precision for quantification strategy, and RT-dependent mode for Cross-run normalization. A library was generated using Smart profiling set up. The primary output file from DIA-NN was further filtered at 1% FDR, and LFQ intensity was calculated using the DIAgui package at 1% q-value (2).

Statistical analysis was conducted using the Perseus program (version 1.6.15.0) from the MaxQuant environment (<http://www.maxquant.org>). Quantifiable proteins were defined as those detected in above 70% of samples in one condition or more. Missing values were replaced using data imputation by randomly selecting from a normal distribution centered on the lower edge of the intensity values that simulates signals of low abundant proteins using default parameters (a downshift of 1.8 standard deviation and a width of 0.3 of the original distribution). To determine whether a given detected protein was specifically differential, a two-sample t-test was performed using permutation-based FDR control at 0.05 and employing 250 permutations. The *p*-value was adjusted using a scaling factor s0 with a value of 1. Analysis was performed on biological triplicates, each injected twice on mass spectrometers.

*2-Biotinylated proteome:* Protein samples were prepared in a similar way to the whole proteome analysis. The final digest was analyzed by liquid chromatography (LC)-tandem MS (MS/MS) using a Q Exactive Plus Hybrid Quadrupole-Orbitrap online with a nanoLC Ultimate 3,000 chromatography system (ThermoFisher Scientific™, San Jose, CA). Peptides were ionized in the EASYSpray source, with a spray voltage set at 1.9 kV and the capillary temperature at 250°C. All samples were measured in a data-dependent acquisition mode. Peptide masses were measured in a survey full scan (scan range 375-1,500 m/z, with 70 K FWHM resolution at m/z=400, target AGC value of 3.00×10^6^ and maximum injection time of 100 ms), and the 10 most intense data-dependent precursor ions were successively fragmented in the HCD cell and measured in Orbitrap (normalized collision energy of 25%, activation time of 10 ms, target AGC value of 1.00×10^5^, intensity threshold 1.00×10^4^ maximum injection time 100 ms, isolation window 2 *m/z*, 17.5 K FWHM resolution, scan range 200 to 2000 *m/z*). Dynamic exclusion was implemented with a repeat count of 1 and exclusion duration of 20 s.

Relative intensity-based label-free quantification (LFQ) was processed using the MaxLFQ algorithm available on the MaxQuant computational proteomics platform, version 1.6.3.4. Analysis was performed on three biological replicates, each injected twice into mass spectrometers. The acquired raw LC Orbitrap MS data underwent initial processing using the integrated Andromeda search engine. Spectra were searched against the *M. abcessus* database (UP000007137) extracted from UniProt on March 10, 2021, containing 4,940 entries. The false discovery rate (FDR) at the peptide and protein levels was set to 1% and determined by searching a reverse database. For protein grouping, all proteins that could not be distinguished based on their identified peptides were assembled into a single entry according to the MaxQuant rules. Statistical analysis was carried out in a similar way to the whole proteome analysis.

*Lipid extraction and TLC analysis*.

Cultures underwent centrifugation for 15 min at 4,000 *g* and were washed twice with water. Pellets were boiled at 95 °C for 10 min, lyophilized overnight, and weighted to determine the exact dry weight of mycobacterial cells. Apolar lipids were extracted according to a previously described method (3). Briefly, 2 mL of MeOH-0.3% NaCl (10:1, *v/v*) was added per 10 mg of dry extract. This saline-MeOH solution containing the bacterial dry extract was mixed for 15 min with 1 mL petroleum ether in Pyrex^®^ tubes at room temperature using a tube rotator. After centrifugation at 3,000 *g* for 5 min, the upper organic layer was transferred to a fresh tube. This step was repeated twice. The combined organic layers containing apolar lipids were transferred to a pre-weighed vial, and the solvent was evaporated to dryness under a nitrogen stream. Finally, the resulting dry apolar lipid residue was re-suspended in dichloromethane.

The extracted lipids were analyzed by thin-layer chromatography (TLC) using glass TLC plates (TLC Silica Gel 60 F_254_, Merck). Following eluent migration (*i.e*., petroleum ether (40-60 °C fraction) / diethyl ether 90:10 (*v*/*v)* for non-polar lipid and Heptane/ diethyl ether/ formic acid 55:45:1 (v/v/v) for acylglycerols), the plates were dried at room temperature for 10 min, and then sprayed with a cupric acetate-orthophosphoric acid solution. This solution was prepared by mixing a saturated aqueous solution of cupric acetate with 85% phosphoric acid in a 1-to-1 volume ratio, followed by heating at 140 °C in an oven for 5-10 min.

References

1. Frankenfield AM, Ni J, Ahmed M, Hao L. 2022. Protein Contaminants Matter: Building Universal Protein Contaminant Libraries for DDA and DIA Proteomics. J Proteome Res 21:2104-2113.

2. Gerault MA, Camoin L, Granjeaud S. 2024. DIAgui: a Shiny application to process the output from DIA-NN. Bioinform Adv 4:vbae001.

3. Santucci P, Johansen MD, Point V, Poncin I, Viljoen A, Cavalier JF, Kremer L, Canaan S. 2019. Nitrogen deprivation induces triacylglycerol accumulation, drug tolerance and hypervirulence in mycobacteria. Sci Rep 9:019-45164.

**Supplementary figures**


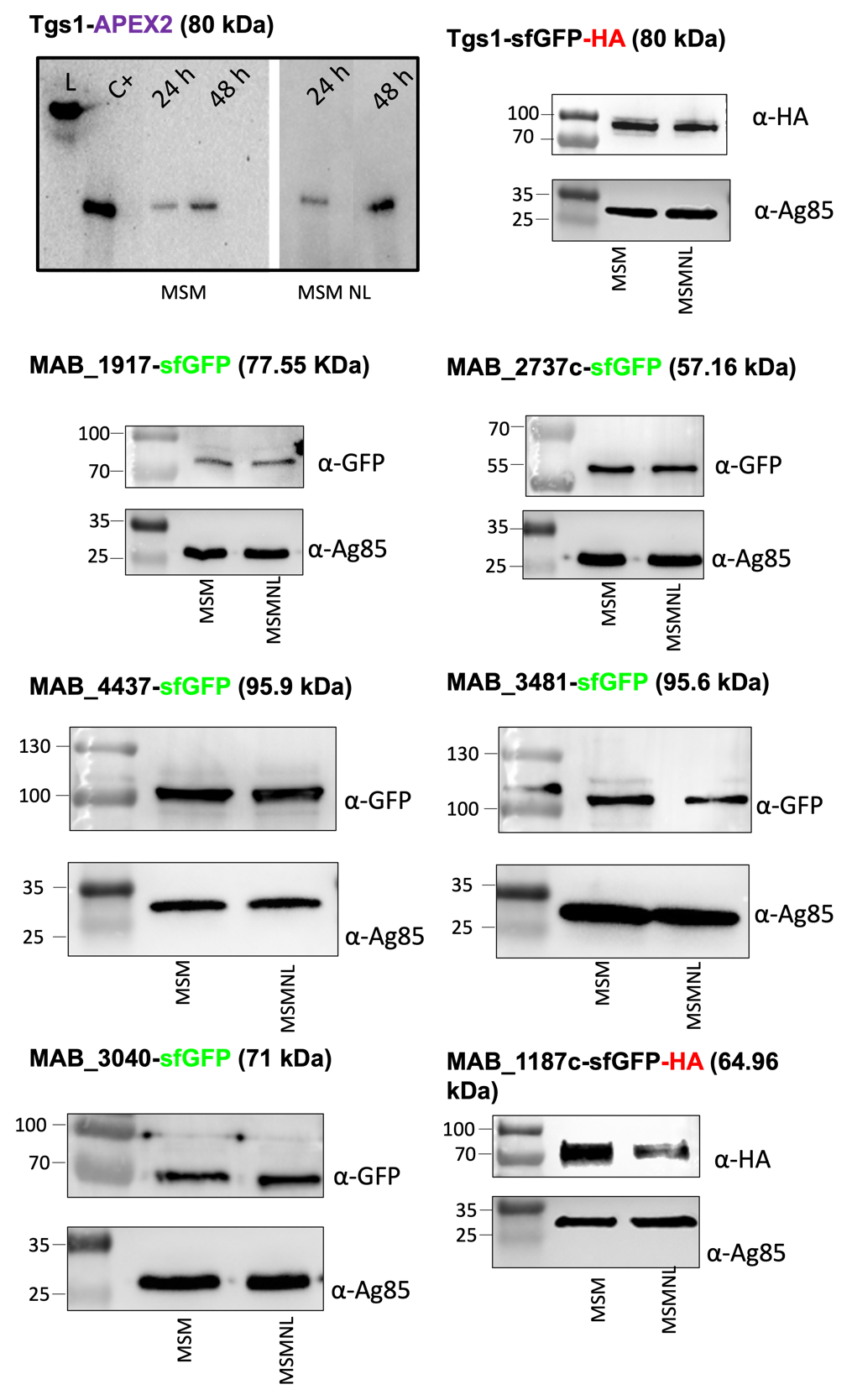


**MAB_1187c-sfGFP-HA (64.96 kDa)**

**Figure S1:** Validation of the sfGFP fusion to each selected protein. Protein expression pattern of each fusion in (WT) in MSM NL and MSM at the specific identification time. Western blotting using anti-APEX2-tag, anti-HA-tag, anti-GFP-tag primary antibodies attest for the expression of APEX2-, HA-, sfGFP-tagged protein in the strains transformed with pMV306. Note: Some proteins (Tgs1 and MAB_1187c) were weakly expressed, so that they were double tagged with GFP and HA since the HA detection is more sensitive than sfGFP detection by Western blotting. Twenty micrograms of proteins were deposited per each well.


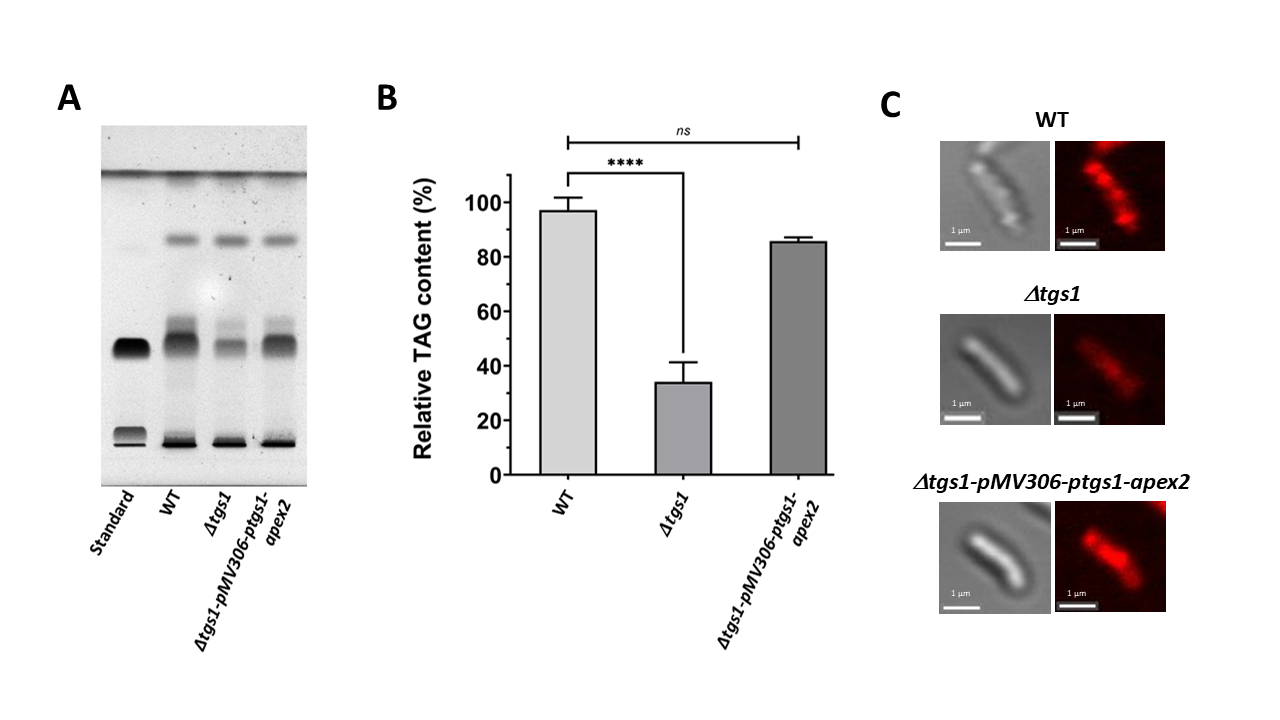


**Figure S2: Tgs1-APEX2 fusion protein is functional.** **(A)** *M. abscessus* strains were grown in MSM NL medium, harvested after 48 hrs, lyophilized, and equal amounts of dry cells were used for apolar lipid extraction. TAG levels from WT, Δ*tgs1*, and Δ*tgs1 pMV306-ptgs1-apex2* complemented strains were analyzed by TLC, with triolein and oleic acid used as standards for TAG and FA, respectively. The TLC plate is representative of two independent experiments. **(B)** Densitometric analysis of TAG levels from TLC data is expressed as mean values ± SD of two independent experiments, with WT levels set at 100%. Statistical comparisons of TAG band intensities were conducted using a one-way ANOVA test (*p* < 0.05). **(C)** Fluorescence microscopy images showing Nile red (559/635 nm) from WT, Δ*tgs1*, and Δ*tgs1 pMV306-ptgs1-apex2* complemented strains grown in MSM NL medium. Fixed samples were stored at 4 °C until Nile-Red staining was performed.


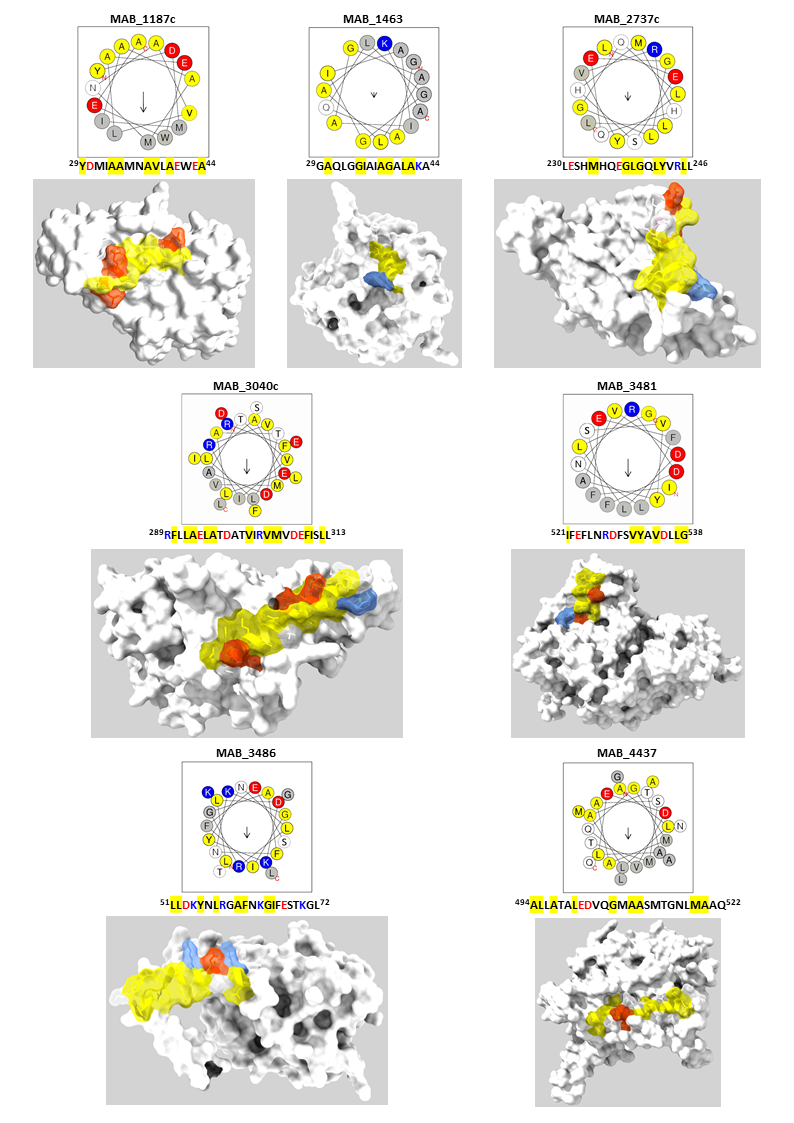


**Figure S3:** Identification and localization of amphipathic helices represented as helical wheel diagrams (top) and their positions on AlphaFold-predicted models (above) from multiple selected proteins in *M. abscessus*. The arrow indicates the angle of the mean hydrophobic moment pointing toward the hydrophobic face of the amphipathic helix, represented in yellow.

**
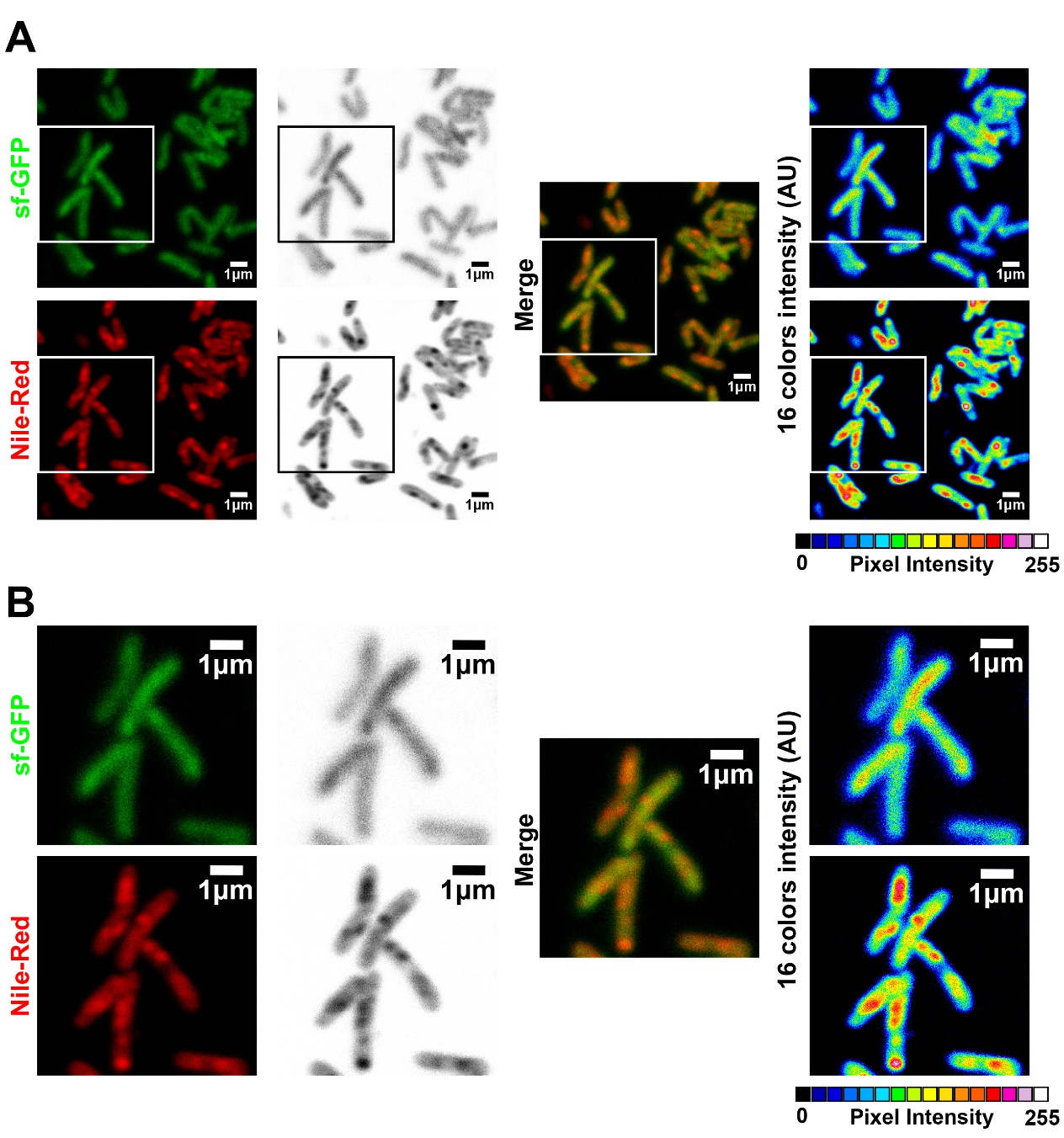
**

**Figure S4: sf-GFP does not localize with ILIs under nitrogen-limiting conditions.** *M. abscessus* harboring the pMV261-*sfGFP* plasmid was grown in MSM NL medium containing 250 µg/mL kanamycin for 48 hrs. Cells were fixed with 4% PFA for 1 h at room temperature and washed twice with PBS. Fixed samples were stored at 4 °C until Nile-Red staining was performed. A stained bacterial suspension (5 μL) was spotted between a coverslip of 170 μm thickness and a freshly prepared 1.5% agarose-PBS pad (~ 1-2 cm^2^). Bacteria were analyzed using snapshot imaging at room temperature with a Leica SP5 laser scanning confocal microscope (Leica Biosystems). Image acquisition was performed with an HC PL APO CS2 63×/1.40 oil objective, capturing 1,024 x 1,024 pixel images. Argon 488 nm and diode-pumped solid-state 561 nm lasers were used for excitation. Emission signals were collected at λ_em_ ~ 500-550 nm for sfGFP and λ_em_ ~585-685 nm for Nile-Red. A single Z-plane was acquired for each field. Individual raw fluorescence images were exported as TIFF files and analyzed using open-source software such as Fiji/ImageJ software (https://imagej.net/software/fiji/). **(A)** Fluorescence imaging of PFA-fixed *M. abscessus* carrying the pMV261-*sfGFP* plasmid. Cells were imaged in both the green fluorescent channel (top panel; shown in green and in grayscale) and the red fluorescent channel (bottom panel; shown in red and in grayscale). A merged micrograph is displayed at the center of the figure. Each individual channel is also represented as 16-colors scaled images on the right (0-255 grayscale units) to highlight ILI and sfGFP distribution profiles. Scale bars represent 1 µm in both X and Y dimensions. **(B)** High magnification views of the zoomed-in regions from the images displayed in **(A)**.

**
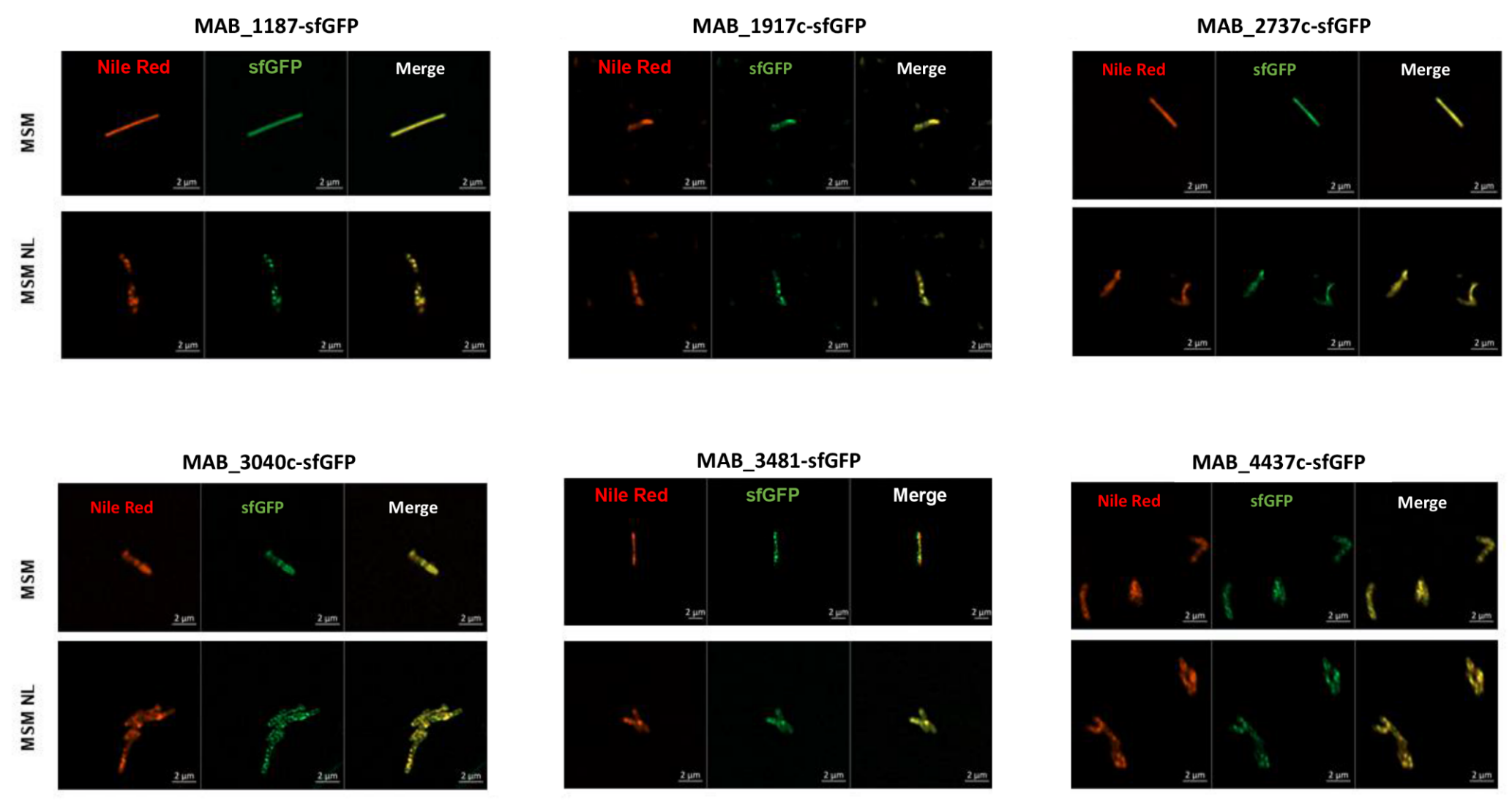
**

**Figure S5:** Colocalization of each selected IAP with ILI. *M. abscessus* was transformed with pMV306-*gene-sfGFP* and grown in both MSM and MSM NL. Bacteria were fixed with 2% PFA, stained with Nile Red and observed by fluorescence microscopy. All the strains expressing each sfGFP-tagged protein strains were grown for 24h except for MAB_3481-GFP which was grown for 48h.


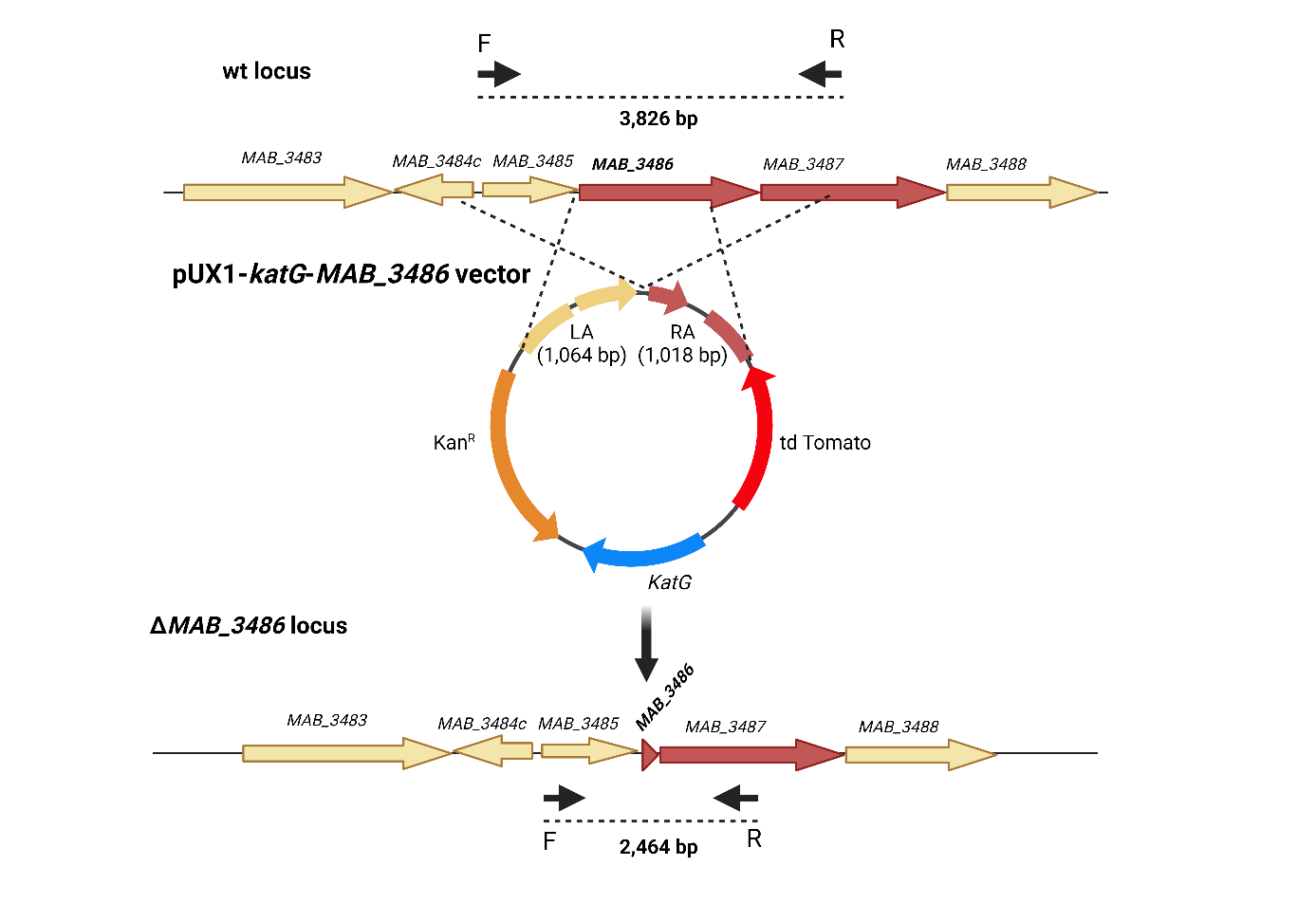


**Figure S6**: Unlabeled deletion strategy for the deletion of *MAB_3486* in *M. abscessus*. *MAB_3486* is located between *MAB_3485* and *MAB_3487*. The pUX1-*katG*-*MAB_3486* plasmid was generated to remove *MAB_3486* by double homologous recombination. The DNA sequence of the left arm (LA) of *MAB_3486* (1,064 bp) was amplified by PCR and cloned into the PacI and MfeI sites of pUX1-*katG*. The DNA sequence of the right arm (RA) of *MAB_3486* (1,018 bp) was amplified by PCR and subcloned into the EcoRI and NheI sites of pUX1-*katG*. The resulting suicide plasmid (lacking motifs for episomal replication or mobile elements promoting chromosomal integration) was used to transform *M. abscessus*, replicating only by homologous recombination between the cloned sequences and their chromosomal homologous sequences. The first recombination events were selected in the presence of kanamycin. Single red fluorescent tdTomato-expressing clones were subjected to the second round of recombination selected with isoniazid and analyzed for double crossover phenotypes, *i.e.* loss of red fluorescence, sensitivity to kanamycin and resistance to isoniazid. The dashed lines represent the size of the expected PCR products in *M. abscessus* wild-type (WT) and Δ*MAB_3486*. Black arrows represent the forward (F) and reverse (R) primers used for PCR analysis.


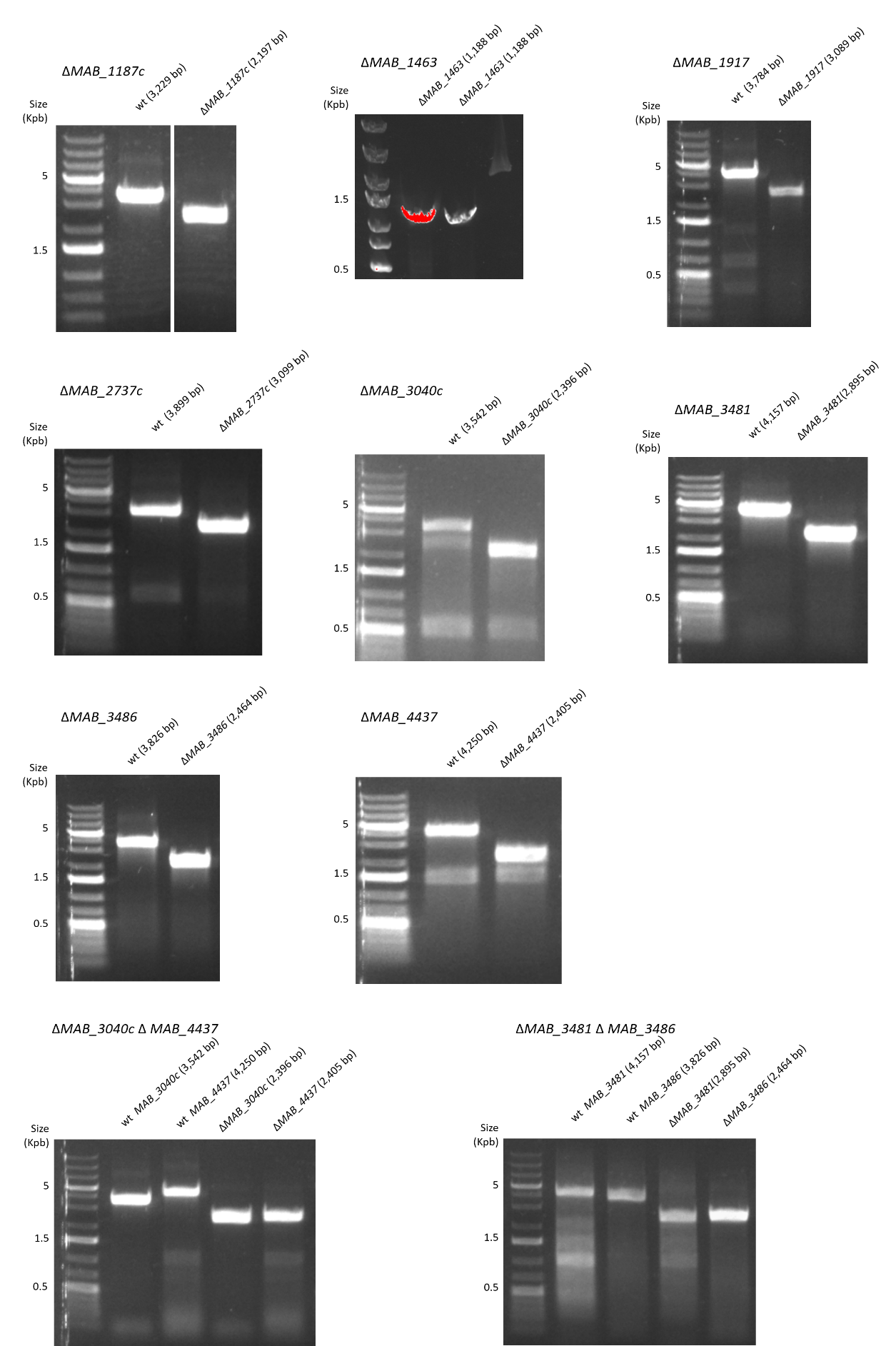

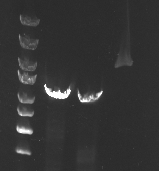


**Figure S7:** Genetic validation of each *M. abscessus* deletion mutant. PCR profile confirming the proper gene deletion of the 10 selected targets. PCR products were amplified from each mutant genomic DNA compared to the WT genome.


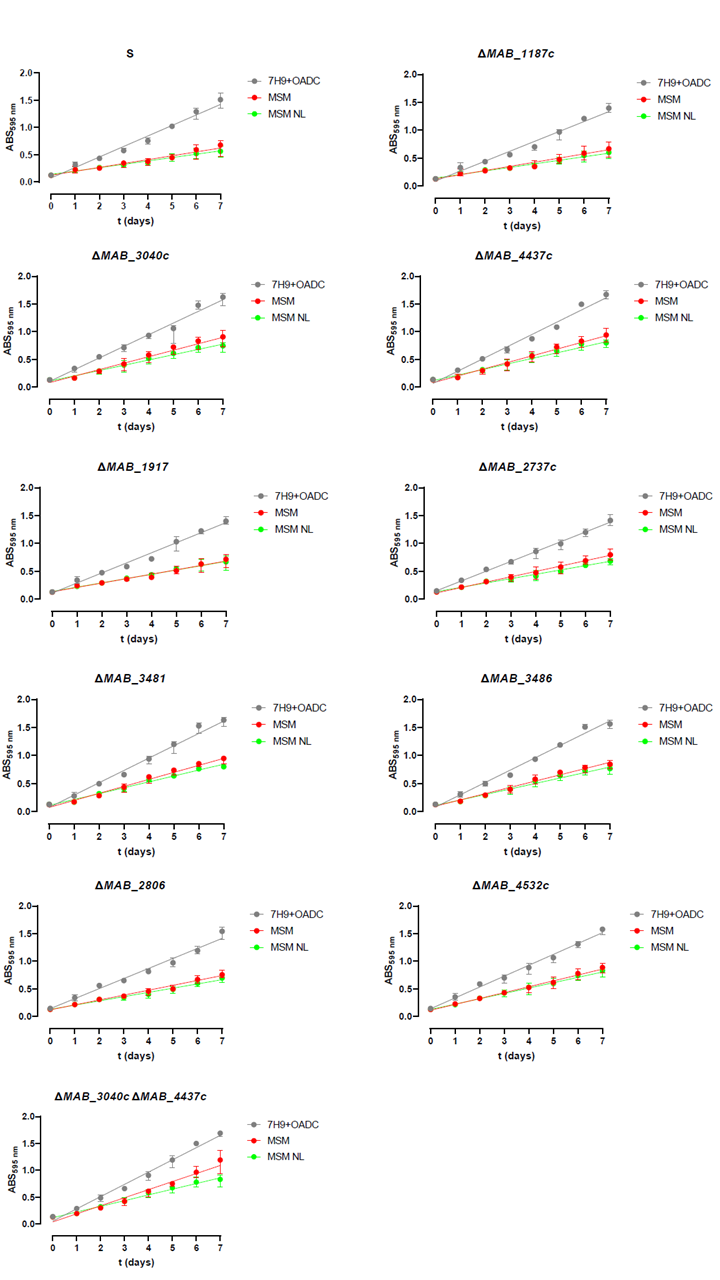


**Figure S8:** Growth curves for the deletion mutants**.** Each deleted strain was grown in 7H9 OADC supplemented with 0.025% tyloxapol (grey), MSM (red), and MSM NL (green) in flat-bottom 96-wells plate for 7 days. Absorbance was measured at 595nm every 24h. Error bars represent standard deviation from three biological replicates. S: smooth morphotype.


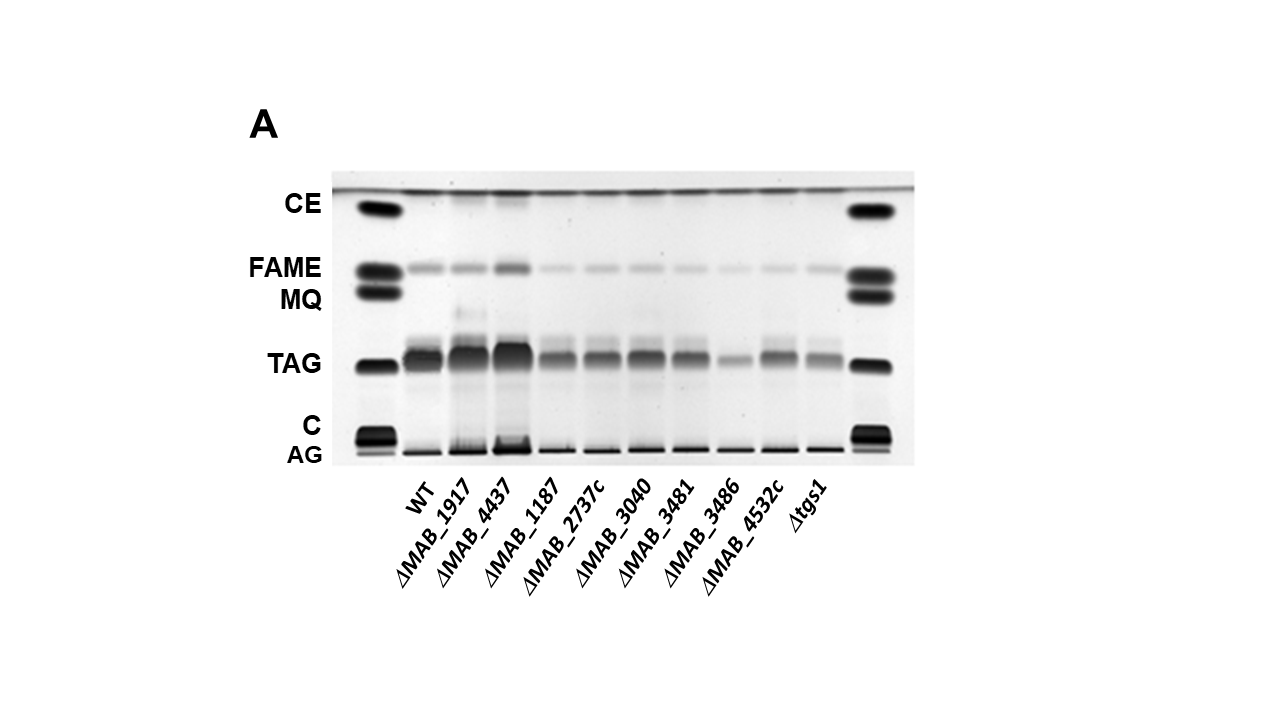

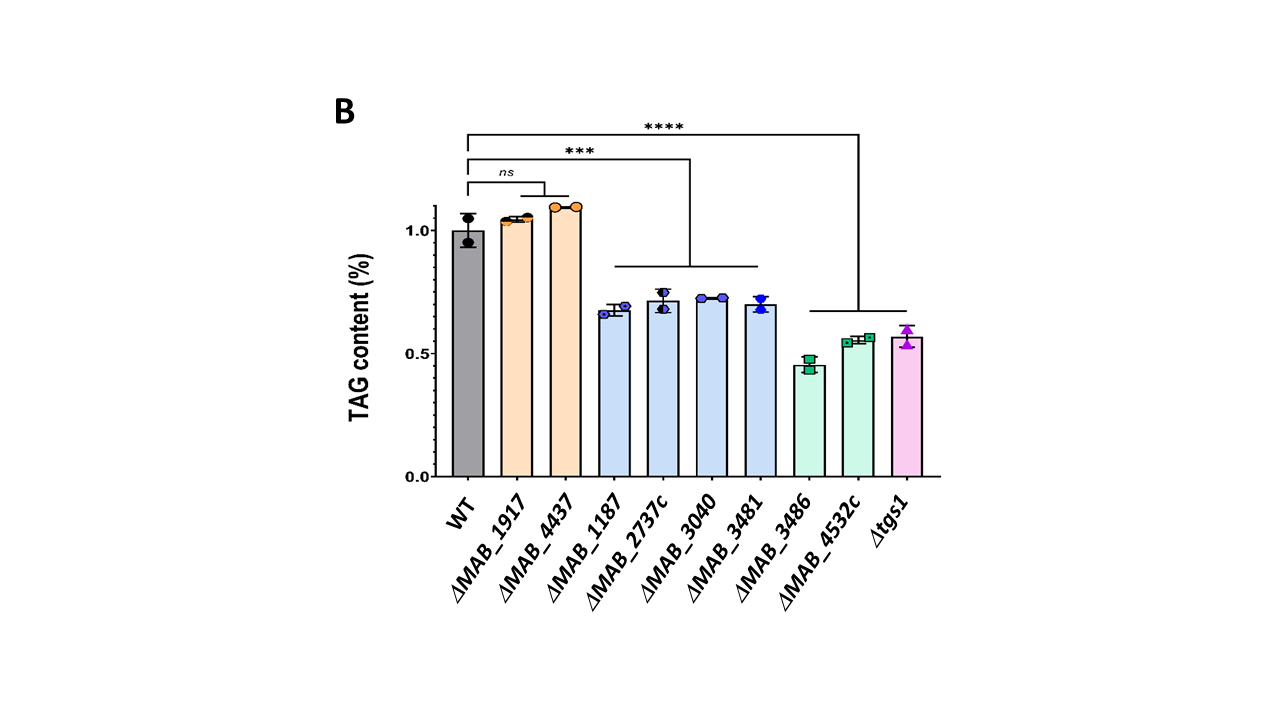


**Figure S9:** Apolar lipid profile in IAP deletion genes. *M. abscessus* deletion mutants were grown in MSM NL. Cultures were collected after a 48h incubation period, lyophilized, and equal amounts of dry cells used for apolar lipid extraction. **(A)** TAG level from each culture were analyzed by TLC. **(B)** Results from densitometry are expressed as mean values ± SD of at least two biologically independent experiments, and reported as relative values using the TAG levels of *M. abscessus* WT in the MSM NL as 100%. Statistical significance was assessed with one-way ANOVA followed by Tukey’s multiple comparisons post-hoc test using Prism 8.0 (GraphPad, Inc): *** *p*-value <0.001; **** *p*-value <0.0001; *ns*, not significant (*p*-value >0.05). C: Cholesterol; CE: Cholesterol Ester; FA: Fatty Acid; FAME: Fatty Acid Methyl Esters; MQ: Mena Quinone; TAG: Triacylglycerol.

**
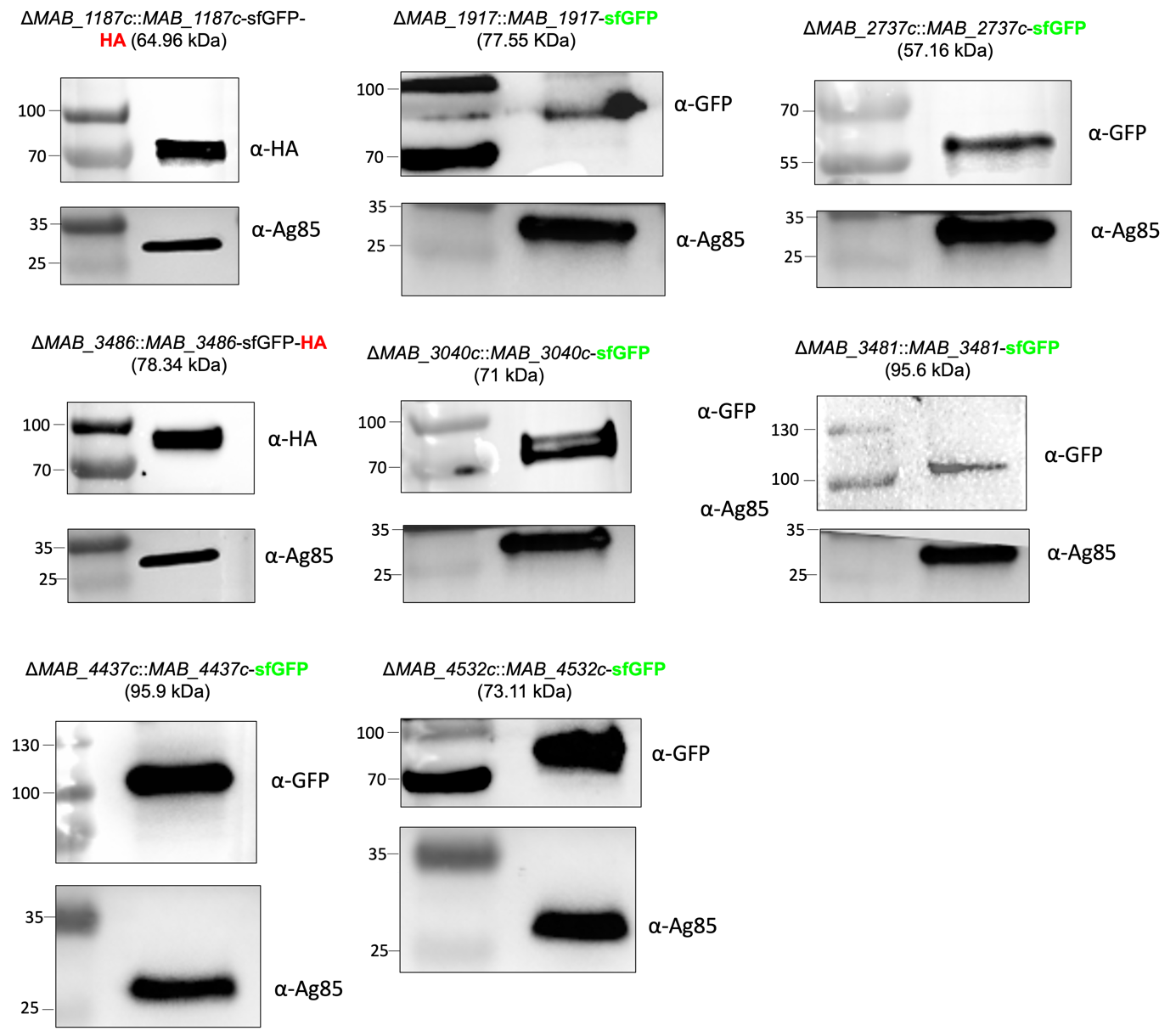
**

**Figure S10:** **Validation of sfGFP-tagged complementation in individual *M. abscessus* mutants.** The expression of sfGFP- or HA-tagged proteins in each mutant strain was evaluated in MSM. A total of twenty micrograms of total protein was loaded into each well for analysis.

**
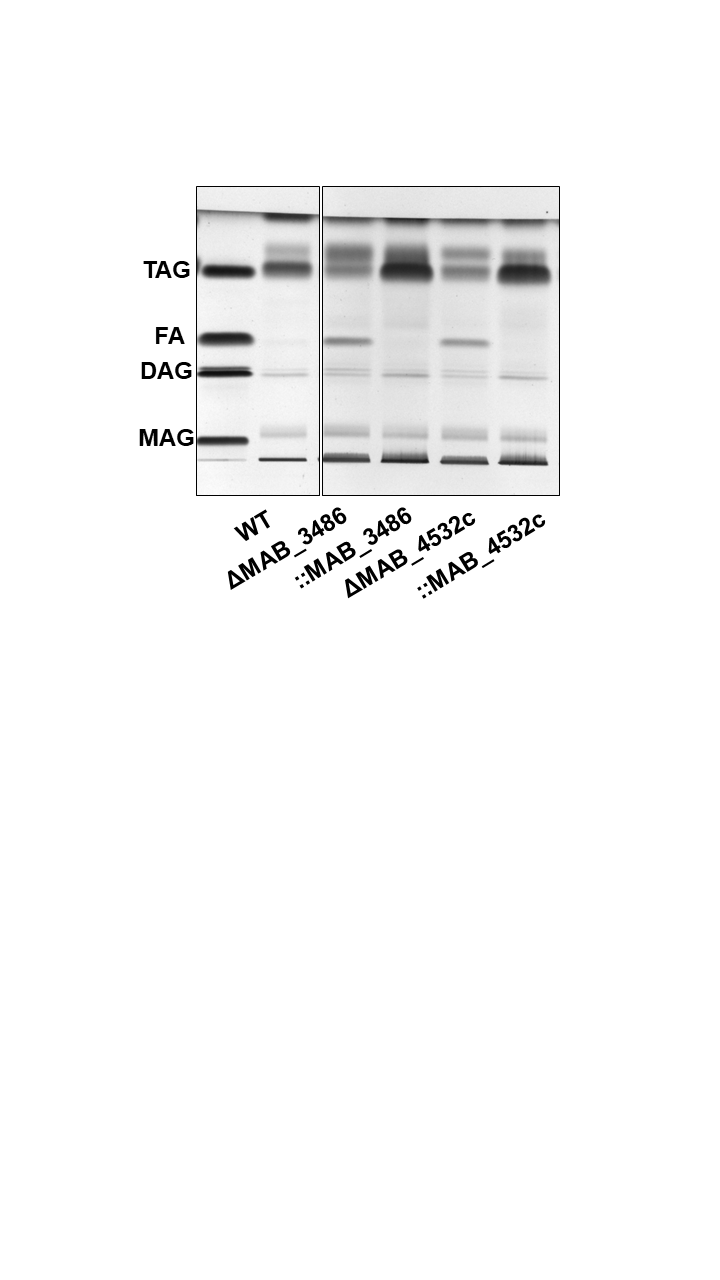
**

**Figure S11: Acylglycerol migration profile** in IAP deleted mutants. *M. abscessus* deletion mutants were grown in MSM NL. Cultures were collected after a 48h incubation period, lyophilized, and equal amounts of dry cells used for apolar lipid extraction. Lipids were separated by TLC. TAG: Triacylglycerol; FA: Fatty Acid; DAG: diacylglycerol; MAG: monoacylglycerol.
